# Musashi and Plasticity of *Xenopus* and Axolotl Spinal Cord Ependymal Cells

**DOI:** 10.3389/fncel.2018.00045

**Published:** 2018-02-27

**Authors:** Ellen A. G. Chernoff, Kazuna Sato, Hai V. N. Salfity, Deborah A. Sarria, Teri Belecky-Adams

**Affiliations:** Department of Biology, Indiana University–Purdue University Indianapolis, Indianapolis, IN, United States

**Keywords:** ependymal cells, musashi-1, musashi-2, spinal cord regeneration, Axolotl regeneration, *Xenopus* regeneration

## Abstract

The differentiated state of spinal cord ependymal cells in regeneration-competent amphibians varies between a constitutively active state in what is essentially a developing organism, the tadpole of the frog *Xenopus laevis*, and a quiescent, activatable state in a slowly growing adult salamander *Ambystoma mexicanum*, the Axolotl. Ependymal cells are epithelial in intact spinal cord of all vertebrates. After transection, body region ependymal epithelium in both *Xenopus* and the Axolotl disorganizes for regenerative outgrowth (gap replacement). Injury-reactive ependymal cells serve as a stem/progenitor cell population in regeneration and reconstruct the central canal. Expression patterns of mRNA and protein for the stem/progenitor cell-maintenance Notch signaling pathway mRNA-binding protein *Musashi* (msi) change with life stage and regeneration competence. Msi-1 is missing (immunohistochemistry), or at very low levels (polymerase chain reaction, PCR), in both intact regeneration-competent adult Axolotl cord and intact non-regeneration-competent *Xenopus* tadpole (Nieuwkoop and Faber stage 62+, NF 62+). The critical correlation for successful regeneration is *msi-1* expression/upregulation after injury in the ependymal outgrowth and stump-region ependymal cells. *msi-1* and *msi-2* isoforms were cloned for the Axolotl as well as previously unknown isoforms of *Xenopus msi-2*. Intact *Xenopus* spinal cord ependymal cells show a loss of *msi-1* expression between regeneration-competent (NF 50–53) and non-regenerating stages (NF 62+) and in post-metamorphosis froglets, while *msi-2* displays a lower molecular weight isoform in non-regenerating cord. In the Axolotl, embryos and juveniles maintain Msi-1 expression in the intact cord. In the adult Axolotl, Msi-1 is absent, but upregulates after injury. Msi-2 levels are more variable among Axolotl life stages: rising between late tailbud embryos and juveniles and decreasing in adult cord. Cultures of regeneration-competent *Xenopus* tadpole cord and injury-responsive adult Axolotl cord ependymal cells showed an identical growth factor response. Epidermal growth factor (EGF) maintains mesenchymal outgrowth *in vitro*, the cells are proliferative and maintain *msi-1* expression. Non-regeneration competent *Xenopus* ependymal cells, NF 62+, failed to attach or grow well in EGF+ medium. Ependymal Msi-1 expression *in vivo* and *in vitro* is a strong indicator of regeneration competence in the amphibian spinal cord.

## Introduction

In all vertebrates, the ependymal cells (ependymoglia) that line the central canal of the spinal cord play essential roles in normal spinal cord structure and physiology (rev. Oksche and Ueck, 1976; Reichenbach and Wolberg, 2013; Jiménez et al., 2014; Pannese, 2015; Moore, 2016). Ependymal cells participate in the spinal cord lesion site response in mammals and represent a clinical target in treating spinal cord injury (SCI) (Mothe and Tator, 2005; Horky et al., 2006; Meletis et al., 2008; Barnabé-Heider et al., 2010; rev. Panayiotou and Malas, 2013; Lacroix et al., 2014; Li et al., 2016). However, the ependymal response in amphibians is more complete and beneficial after SCI.

**FIGURE 1:**
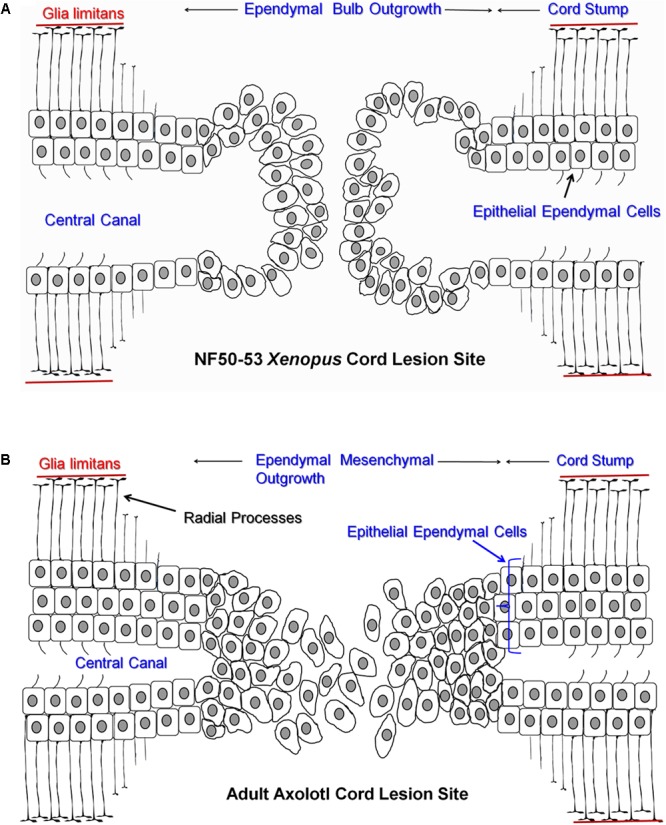
Cartoon representing ependymal outgrowth from cranial (Left) and caudal (Right) stumps of regenerating *Xenopus* and Axolotl spinal cord. **(A)** Regenerating NF 50–53 *Xenopus* tadpole cord showing gap regeneration with ciliated epithelial ependymal cells in the stump and the bulb-like ependymal outgrowth. **(B)** Regenerating adult Axolotl gap regeneration with mesenchymal ependymal outgrowth and several layers (bracket) of epithelial ependymal cells in the stump. The *glia limitans*, the basal lamina of the cord, is represented as a red line adjacent to ependymal endfeet. All other cell types and extracellular matrix were omitted to simplify the representation.

The ependymal response, and the extent and mechanism of regeneration, is not uniform across all amphibians and all stages of life. There are strong differences in ependymal behavior and regeneration capacity between anuran amphibians (frogs, toads) and urodele/caudate amphibians (salamanders, newts). Anurans regenerate only as young tadpoles while urodeles are strong cord regenerators through adulthood (Dent, 1962; Mitashov and Maliovanova, 1982). In addition, the ependymal response changes with life stage even in urodele amphibians (rev. Chernoff et al., 2003; Becker and Becker, 2015). The present paper will compare *Xenopus laevis* (the African Clawed Frog) tadpoles stages NF 50–54 (Nieuwkoop and Faber, 1956; regeneration competent) vs. NF 60–64 (regeneration incompetent) and embryonic, juvenile and adult salamanders of the species *Ambystoma mexicanum* (the Mexican Salamander or Axolotl). **Figure 1** shows a cartoon representation of the cellular outgrowth phase of gap regeneration (regeneration between stumps of transected cord) emphasizing the bulb-like nature of ependymal outgrowth in *Xenopus* (**Figure 1A**) and the mesenchymal ependymal outgrowth in the Axolotl (**Figure 1B**). The extent to which ependymal epithelium disorganizes during regeneration is species and location specific (Clarke and Ferretti, 1998; Chernoff et al., 2003; Gargioli and Slack, 2004; Zukor et al., 2011).

In anuran amphibians, such as *Xenopus*, regenerative capacity in the central nervous system (CNS) is one of many processes affected by progress toward and through metamorphosis (Shi, 2000). The spinal cord will regenerate when it is transected in young tadpoles, a stage of life in which the animals are still developing rapidly (Holder et al., 1989; Beattie et al., 1990; rev. Diaz Quiroz and Echeverri, 2013; Lee-Liu et al., 2013; Becker and Becker, 2015). Aside from a transient refractory period at stages NF 45–47, it is generally agreed that *Xenopus* regeneration fails permanently when the spinal cords of frogs and toads are lesioned at the end of metamorphic climax and that tadpoles lesioned during the period permissive for regeneration must continue to grow and progress toward metamorphosis in order to achieve complete regeneration (Forehand and Farel, 1982; Beattie et al., 1990; Beck et al., 2003). The precise stage at which anuran spinal cord regeneration fails depends on the species, the location and type of lesion, and the axonal tracts examined (Forehand and Farel, 1982; Clarke et al., 1986; Holder et al., 1989; Beattie et al., 1990).

Urodele amphibians, such as the Axolotl, can regenerate lesioned spinal cord through axonal sprouting from uninjured neurons, and regrowth of axons is associated with ependymal processes/channels and the basal lamina produced by the endfeet of ependymal cell processes. Neurons can be recruited into the regenerating cord from regions adjacent to the lesion site, and new neurogenesis from ependymal cells with neural stem cell properties also occurs (Egar and Singer, 1972; Chernoff et al., 2002, 2003; Ferretti et al., 2003; Mchedlishvili et al., 2007; Becker and Becker, 2015). Additional ependymal roles in cord regeneration include the interaction of reactive ependymal cells with the reactive meningeal connective tissue to prevent fibrotic/meningeal scar formation, including production of matrix metalloproteinases to remove extracellular matrix material (Chernoff et al., 2000; Zukor et al., 2011). Not all of these regenerative processes have been documented at every stage of the urodele life cycle. The reactive body region/non-tail ependymal cells grow into the lesion site as either an epithelioid bulb (newts) or a mesenchymal mass (Axolotl) (Chernoff et al., 2003; rev. Zukor et al., 2011). In Axolotls, as in *Xenopus* tadpoles (shown histologically Muñoz et al., 2015), regeneration in the larval stage reflects the ongoing rapid development of the animal and there are significant differences between juveniles and adults, as well (rev. Chernoff et al., 2003). Axolotls are aquatic, neotenic animals (they fail to undergo complete metamorphosis), but CNS regeneration in the Axolotl and other urodeles is not dependent on the neotenic state (Chernoff et al., 2003; Amamoto et al., 2016).

While it is recognized that amphibian ependymal cells can act as neural stem cells, there are no unique neural stem cell markers in any species that are not present in other tissues and used for other biological processes. Combinations of markers, such as intermediate filament proteins, including nestin and vimentin, and ciliary proteins, have been used, but their presence and distribution in the CNS is region and species specific (rev. in Chernoff et al., 2003; Ren et al., 2017). There is no clear role for intermediate filaments in the regeneration process and, as Ren et al. (2017) point out, there are other ciliated cells in the CNS. The stem cell marker Sox2 has been examined in *Xenopus* tadpole tail regeneration and Axolotl larva tail regeneration. Muñoz et al. (2015) showed that neurogenesis markers increased after injury only in regeneration-competent tadpoles and were dependent on expression of the stem cell-related Sox2/3 gene. CRISPR genomic deletion of Sox2 in the Axolotl resulted in failure of ependymal cell proliferation at the tail amputation site in larval animals (Fei et al., 2014). Some Sox2+ ependymal cells exist in adult Axolotl cord (Tazaki et al., 2017).

Another useful marker is Musashi (Msi), an mRNA-binding protein which is a member of the Notch signaling pathway. Msi expression has been identified in ventricular zone or sub-ventricular zone neural stem cells and neural progenitor cells in mammals (Sakakibara et al., 1996; Kaneko et al., 2000). Disruption of Notch signaling can cause premature differentiation of Sox-2-positive neural progenitor cells (Ehm et al., 2010). Numb is an inhibitor of intracellular Notch processing, and *Numb* mRNA is bound by Msi protein, which prevents *Numb* mRNA translation. Activation or maintenance of Notch signaling by inhibition of *Numb* translation suppresses premature onset of neuronal differentiation, maintaining neural stem or progenitor status (Imai et al., 2001). Overexpression of Msi-1 can activate Notch signaling (Imai et al., 2001). Notch pathway roles in developing nervous system also include effects on proliferation and apoptosis (rev. Faigle and Song, 2013). These observations make Msi, particularly Msi-1, a marker of interest in spinal cord regeneration.

Musashi-1 and -2 were first identified as Nrp-1 and Xrp-1, respectively, by Peter Good’s laboratory in *Xenopus* ependymal cells (Richter et al., 1990). In vertebrates those genes became known as Musashi-1, -2 following the identification of *Drosophila* Musashi (d-msi) and that convention is followed in this paper (Nakamura et al., 1994).

The ependymal response after SCI is not uniform between the amphibian orders anura and urodela, in different species within one order, at different stages of the life cycle, or even in tail vs. body spinal cord within one species. The ependymal cells must be physiologically functional in all amphibian cord, but their differentiated state varies. It should not be surprising, then, that the nature of expression of members of the Msi family of stem cell markers varies with life stage and ependymal behavior. The studies described here emphasize that active expression of Msi-1 is a useful marker of the plasticity necessary for participation of amphibian ependymal cells in spinal cord regeneration.

The expression of known and newly cloned forms of Msi-1 and Msi-2 are examined here. Comparisons are made between: intact vs. regenerating cord; developing, juvenile and adult cord; and anuran vs. urodele cord ependymal cells. Section *in situ* hybridization, RT-PCR and antibody localization are used to follow life stage and regeneration-related changes in expression of Msi-1 and Msi-2. Msi-1 expression is viewed along with that of the dorsal-ventral axis (DV) patterning gene sonic hedgehog (Shh) in *Xenopus* and the Axolotl in order to indicate the relationship of the expression of this stem/progenitor cell with cord DV axis maintenance or repatterning studied by others. Disrupting either Notch signaling or DV patterning genes like Shh or bone morphogenetic proteins inhibits larval or tadpole regeneration, respectively, though DV patterning genes may act upstream of Notch (Beck et al., 2003; Schnapp et al., 2005). In the adult Axolotl, where Msi-1 must be re-activated, the Msi-1 expression pattern is further compared with the presence and loss of the intermediate filament protein glial fibrillary acidic protein (GFAP), as an indicator of the organizational changes that accompany tissue reorganization and Msi-1 reexpression. Antibody localization of proliferative cell nuclear antigen (PCNA) is used in analysis of proliferative capacity *in vivo* and is compared with regeneration-competence and Msi-1 expression.

Despite differences in the initial presence or absence of Msi-1 at the time of injury, or the extent of tissue reorganization *in vivo*, regeneration-competent *Xenopus* tadpole and adult Axolotl ependymal cells share common aspects of cell behavior. *In vitro* studies presented here suggest that there are common features in growth and trophic factor responses in cell outgrowth and reformation of an ependymal epithelium. Epidermal growth factor (EGF) is known to maintain injury-reactive Axolotl ependymal cells in a mesenchymal state *in vitro*, and EGF-containing defined medium is shown here to support mesenchymal growth from *Xenopus* ependymal cells, as well (Chernoff et al., 1990; O’Hara and Chernoff, 1994).

## Materials and Methods

### Surgical Methods

#### *Xenopus*: Animals and Surgery

Animals were obtained from Xenopus Express or Nasco (US locations). Tadpoles were anesthetized in 0.5 g/L concentration of Finquel (tricaine methanesulfonate; Western Chemical, Inc), with 0.2% thimerosal (Sigma), for disinfection, at a sodium bicarbonate-adjusted pH between 7.2 and 7.4. Spinal cord tissue was removed from the dorsal portion of appropriately staged tadpoles with fine forceps and syringe needles and transferred through a series of 73% Leibovitz L-15 medium rinse solutions on ice. Tissue was then either processed for mRNA isolation, sectioning or cell culture as described in subsequent sections.

#### Axolotls: Animals and Surgery

Axolotls were obtained from the Ambystoma Genetic Stock Center, University of Kentucky or bred in our vivarium. Animals were maintained at 23°C in 20% Holtfreter’s salts solution. The transdermal anesthetic Finquel was used in 20% Holtfreter’s, adjusted to pH 7.4 with sodium bicarbonate. Animals were anesthetized in 0.1% Finquel (for adult; >20 cm, 2∼3 years old), 0.025% Finquel (for juveniles; 10∼15 cm, 6 months old). The lesioning procedure is described in detail in Chernoff et al. (1990). Briefly, a skin flap was cut and reflected back. Muscle was removed and a slot cut in the left side of the vertebral arch. Iridectomy scissors were used to transect the cord. The skin flap was replaced and allowed to clot in place. Post-surgically, the lesioned-cord animals were treated in 20% Holtfreter’s solution at 12°C in a BOD incubator for 2 days and kept at 23°C in the vivarium through the regeneration process. All husbandry, surgery, analgesia, and euthanasia was performed following our IUPUI School of Science IACUC approved protocols.

### Molecular Biology Techniques

#### Cloning

Total RNA was isolated with Trizol according to the manufacturer’s instructions (Roche, Indianapolis, IN, United States) and 1 μg of total RNA was used as a template and reverse transcribed with SuperScript III (Invitrogen, Carlsbad, CA, United States) and oligo dT20 primers. PCR of the Msi-1 partial coding sequence was performed using Platinum Taq polymerase High Fidelity (Invitrogen, Carlsbad, CA, United States). The following primers were used.

GCAGCTATATCAGTGCAGCAAG *Xenopus* nrp-1a (Msi-1) isoform forward
CAGGAACACAAGGTGAGGTACAG *Xenopus* nrp-1a (Msi-1) isoform reverse
ACGCGGGGCCCCTCTCCCTGCTCCTCT Axolotl Msi-1 Full-1 forward
CCAGTCGGAGGTTTCTCCTTCTAGAGT Axolotl Msi-1 Full-1 reverse
CGACTCGTGCTGGAGGATTTCTA Axolotl Msi-1 isoform forward
GGAGTGAGCTGCTCTCCTGACAA Axolotl Msi-1 isoform reverse
GAGCCGCCATGGAGGCAGACGGAATT Axolotl Msi2 Full forward
GCAAGTTGAGGGACATCCGATTT Axolotl Msi2 Full reverse
gccgccATGGAGGCAGACGGAATT Axolotl Msi2S ORF-5′ forward
gagCTACTTTTGGCTCCACAGGCC Axolotl Msi2S ORF 3′ reverse

The product was amplified by PCR. The amplimer was directionally cloned into pCR-BluntII-TOPO plasmid vector (Invitrogen, Carlsbad, CA, United States) and all clones were sequenced to verify their identity prior to analyzing expression patterns. Open reading frames and protein sequences were identified and aligned with human, mouse, and *Xenopus laevis* coding sequences using ClustalX^1^.

#### Genbank Accession Information

musashi-like protein 2L [Ambystoma mexicanum]
GenBank: ACS92717.1 Sato, K and Chernoff, EAG
musashi-like protein 2S [Ambystoma mexicanum]
GenBank: ACS92718.1 Sato, K and Chernoff. EAG

#### Semi-Quantitative RT-PCR

Semi-quantitative Reverse Transcription-PCR was performed as defined by Ferre (1992). The length of the transcript, GC content of the primers and transcript define this method. The transcript was examined every five cycles and the resulting amplification curve kinetics were used to determine number of replication cycles. Duplicate sets of tissue from 5 to 6 Axolotls was used per experiment (total = 10 to 12 per experiment). Total RNA was extracted with Trizol (Roche), and treated by RQ-1 RNase-free DNase (Promega). Aliquots of 1 μg total RNA were used as reverse-transcription (RT) templates. Reverse transcription was performed using Super Script III RT (Invitrogen) and oligo dT20 primers. PCR was performed using Platinum Taq polymerase. Primers RT-PCR were as follows:

Axolotl Musashi-1 (Forward; 5′- CAGGCTGGTGTGGACAAGGTTCTGG-3′,
Reverse; 5′- GGGGACATGACCTCCTTTGGCTGAG-3′; 365 bp) at 30 cycles,
Axolotl Musashi-2 (Forward; 5′- CCTGAGCTGGCAGACCTCACCAGATA-3′,
Reverse; 5′- CTTAGGCTGTGCTCTTCGCGGAAAC-3′; 232 bp) at 33 cycles,
Axolotl Shh (Forward; 5′-GCTCTGTGAAAGCAGAGAACTCG-3′,
Reverse; 5′-CGCTCCGTCTCTATCACGTAGAA-3′ 229 bp, at 30 cycles;
Axolotl GAPDH (Forward; 5′- GCTAAGCGTGTGATCATCTCTG-3′,
Reverse; 5′- GTCATGAGACCCTCCACAATG-3′; 182 bp) at 30 cycles.

#### Section and Dish *in Situ* Hybridization

Section *in situ* hybridization was performed as in Sato and Yasugi (1997), a modification of Wilkinson (1992, 1993). Tissues were fixed overnight in 4% paraformaldehyde in PBS at 4°C, rinsed and infiltrated with sucrose to 30% at 4°C, embedded in OCT and cryosectioned. Samples were stored at -80°C. The tissue was cryosectioned and 12 μm sections attached to Vectabond-coated slides (Vector Labs) and dried. Sections were rehydrated in PBS plus 0.01% Tween-20 (PBT) for 5 min, treated with 1 μg/ml proteinase K (Sigma Chemical Company) at 37°C for 5–10 min. Samples were washed three times with PBT, 1 min each, then post-fixed in 4% paraformaldehyde in PBS for 20 min. After post-fixation the sections/samples were rinsed 2× with PBT, 1 min each. Sections were hybridized with digoxigenin-labeled RNA probes (0.3–3 mg/ml) in hybridization buffer (5× SSC, pH 4.5 with 50% formamide, 50 μg/ml yeast total RNA, 50 μg/ml heparin and 1% sodium dodecyl sulfate (SDS) overnight in a humidified box at 70°C. Riboprobes were prepared using full-length cDNA sequence. Hybridized sections were washed twice with 5× SSC (pH 4.5) with 50% formamide and 1% SDS, for 30 min each at 65°C. Samples were then washed three times in 2× SSC (pH 4.5) with 50% formamide for 30 min at 65°C. Three washes were performed with Tris–HCl buffered saline containing 0.05% Tween-20 (TBST) and sections were incubated with 1% blocking reagent (DIG Nucleic Acid Detection Kit: Roche) in TBST. The samples were incubated overnight at 4°C with pre-absorbed alkaline phosphatase conjugated anti-digoxigenin antibody Fab fragment (diluted 1:2000 with 1% heat-inactivated sheep serum in TBST), then washed three times in TBST with 2 nM Levamisole for 20 min each. Samples were incubated for 5 min in NTMT buffer (100 mM Tris–HCL [pH 9.5], 100 mM NaCl, 50 mM MgCl_2_, and 0.1% Tween-20 plus 2 mM Levamisole). Color development was performed in coloring solution NTMT buffer with 0.45 μl/ml nitro blue tetrazolium (NBT, Roche), 3.5 μl/ml 5-bromo-4-chloro-3-indolyl-phosphate (BCIP, Roche and 2 mM Levamisole) in the dark, with periodic microscope examination, until the desired reaction product intensity was obtained. The reaction was stopped by rinsing in 4% paraformaldehyde in PBS several times for 10 min. Samples were mounted in glycerol and coverslipped. For the *Xenopus* dish *in situ* hybridization, the cultures were fixed in 4% paraformaldehyde in 0.1 M phosphate buffer, pH 7.4 at 4°C for 2 h, washed in PBS twice, 15 min each rinse, at 4°C. Section and dish *in situ* samples were mounted in glycerol and coverslipped. Digital photomicrographs were captured using a Nikon E-800 fluorescence/DIC microscope for sections or a Nikon TE-2000 inverted optics microscope for cell cultures, both with a DXM-1200F digital camera (Nikon; Japan).

### Immunohistochemistry

Dissected tissues were fixed in 4% paraformaldehyde in 0.7× (*Xenopus*) or 1× (Axolotl) PBS at 4°C for at least 1 h. Samples from at least five *Xenopus* and three Axolotls at each stage studied were fixed, embedded, sectioned, and examined. The difference in numbers between species reflects the small animal size and small amount of cell outgrowth in *Xenopus*, requiring more samples. The fixative was removed, samples rinsed in PBS and dehydrated in a graded ethanol series. Alcohol dehydration was followed by two xylene rinses, paraffin penetration and paraffin embedding. The sections were cut to 10 μm thickness by microtome and “baked” onto Superfrost/Plus Microscope slide glass (Fisher) at 50°C for 5 h. After deparaffinizing and rinsing with PBT (phosphate-buffered saline, PBS, plus 0.1% Tween-20), the sections were boiled in a microwave in 0.01 M citrate buffer (pH 6.0) for ∼1 min, for post-fixation antigen recovery, unless otherwise noted. The sections were then treated with a blocking buffer as follows: PBT with 10% normal goat serum (NGS), diluted 1:1 with Superblock (Pierce Chemical) for proliferating cell nuclear antigen (PCNA), 10% normal donkey serum in PBT buffer, diluted 1:1 in Superblock for Musashi-1 (Msi-1) antibody and 0.5% Blocking Reagent (Roche) in Tris Buffered Saline and Tween (TBST) with 10% NGS (GFAP). The primary antibodies were added in the Tris Buffered Saline (pH 7.5) and incubated overnight at 4°C. After washing with the matching PBST (see blocking step), sections were incubated with Alexa Fluor 594 secondary antibody (1:2000, Invitrogen) for 2 h at room temperature. Msi-1 antibody (R&D Systems) was diluted 1:25, no citrate treatment. PCNA (PC10 clone) monoclonal antibody (Sigma) was diluted 1:3000. Glial Fibrillary Acid Protein (GFAP) rabbit polyclonal (DAKO) was diluted 1:500. After washing with PBT twice, sections were mounted in SlowFade Gold antifade reagent with DAPI as the nuclear counterstain (Invitrogen) and coverslipped. The specimens were observed with a fluorescence microscope.

### Cell Culture

#### *Xenopus* Tissue Isolation for Cell Culture

Tissue was isolated as described in *Xenopus*: Animals and Surgery. The tissue rinse solution contained the following antibiotics at a 1% concentration: Pen-Strep, Fungizone, gentamicin, and amikacin (Sigma). The pH of the tissue rinsing solution was readjusted using 0.1 N NaOH to pH 7.4. When necessary, the tissue was cut into smaller pieces using micro-scissors before transferring to a conical vial for dissociation in 0.5 ml aliquots of 73% *Xenopus* medium. Tissue was dissociated using a flame-narrowed glass pipette. After each dissociation, the cells were examined microscopically and aliquots were combined. Cells were counted on a hemocytometer and seeded at a density of ∼200,000 per 35 mm dish.

#### *Xenopus*: Removal of Non-ependymal Cells from Cultures

Spinal cords were removed from sets of 10–12 tadpoles for each set of cultures. Dissociated cells were evenly divided among three 35 mm culture dishes, and placed in a BOD incubator at 21°C for at least 1 h to allow for maximal cell adhesion. Medium containing unattached cells and debris was drawn off and a 1% antibiotic solution was added to prevent contamination of the cultures. To remove neurons the dish was incubated in calcium/magnesium-free (CMF) Hanks’ Balanced Salts Solution (HBSS, Fisher Scientific) at room temperature for 10 min. The neurons were removed by gentle pipetting under a stereomicroscope, and the result examined on an inverted phase contrast microscope. The CMF-HBSS was replaced with L-15-based medium described above and returned to the incubator. After the first 2 days, antibiotic-free *Xenopus* medium was used. This medium was then changed every other day. Once culture conditions were standardized each experiment/stage studied was repeated three times.

#### *Xenopus* Culture Medium

*Xenopus* culture medium was prepared using a base of 73% Leibovitz L-15 medium (GIBCO) with 10 mM HEPES (4-(2-hydroxyethyl)-1-piperazineethanesulfonic acid). The following components were then added: glucose 10 mM, CaCl_2_ 1 mM, EGF 20 and 5 ng/ml and progesterone 20 nM. Finally a stock solution containing 5 μg/ml insulin, 100 μg/ml transferrin, 100 μM putrescine, and 30 nM selenium was added (Sigma Chemical). The pH of the solution was then adjusted to 7.6 with 1.0 N NaOH. The medium was filter sterilized and stored at 4°C. All medium additives were from Sigma Chemical Inc.

#### Axolotl Tissue Culture

For these experiments sets of four or five animals, matched in age, were lesioned. Explants were isolated following the procedure described in Chernoff et al. (1990) and O’Hara and Chernoff (1994). Two week outgrowth was isolated, freed of clinging meninges and cultured on poly-D-lysine/fibronectin-coated dishes. Explants were divided among three dishes. Each experiment was repeated three times. Culture medium consisted of 97% Leibovitz L-15 medium and the same additives described above for *Xenopus*.

#### Culture Dish Preparation

Thirty-five millimeter polystyrene culture dishes were coated with 100 μg/ml poly-D-lysine made up in HEPES-buffered saline solution, pH 7.4. The dishes were incubated at 37°C for at least 30 min and then rinsed twice with HEPES buffer. Fibronectin (75 μg/ml) was subsequently added to each dish and incubated again at 37°C for an additional 30 min to 1 h and rinsed with HEPES buffer. HEPES buffer solution contained 0.01 M HEPES, 0.01 M KCl and 0.013 M NaCl in water, adjusted to pH 7.4 with 1 N NaOH. The dishes were rinsed with medium prior to addition of cell-containing supernate from the tissue dissociation.

## Results

### Cloning

#### Msi-1 Alignment

**FIGURE 2:**
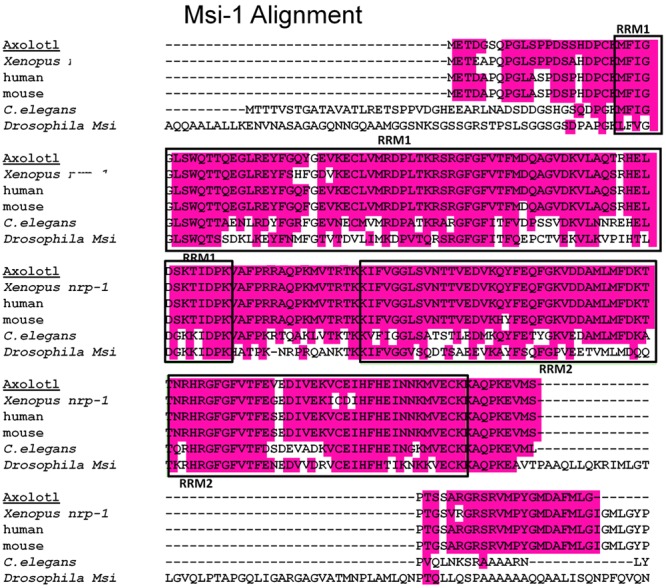
Msi-1 alignment, deduced amino acid sequences. A multispecies alignment including both *Xenopus* and Axolotl is shown. The RNA recognition motifs (RRMs), also known as mRNA-binding domains, are indicated by the boxed areas labeled RRM1 or RRM2. The RRMs are located near the amino terminus of the protein. A 16 amino acid linking region between RRM1 and RRM2 is identical in *Xenopus*, Axolotl, mouse, and human. Amphibian and mammalian RRM sequences all have greater than 90% homology at the amino acid level, details in the Section “Results.”

Axolotl Msi-1 and Msi-2 were cloned as described in the Section “Materials and Methods”. Sequence analysis showed *Xenopus* Msi-1 to be identical to nrp-1 as reported by Richter et al. (1990). A multispecies alignment for Msi-1 is shown at the amino acid (a.a) level in **Figure 2**. *Xenopus* Musashi (published as nrp-1 in Richter et al., 1990), and Axolotl Msi-1 are presented along with mammalian, and invertebrate forms. Homology of the mRNA recognition motifs (RRMs) was high among *Xenopus*, Axolotl, mouse, and human. Both RRM1 and RRM2 were 73 amino acids (deduced a.a. sequences). Between *Xenopus* and mouse or human RRM1 was 93.2% identical and RRM2 was 95.9% identical. Between Axolotl and mouse or human RRM1 homology was 97.3% identical and RRM2 was 98.6% identical. Between *Xenopus* and Axolotl, RRM1 was 93.2% identical and RRM2 was 95.9% identical; the percentage deduced amino acid sequence difference between *Xenopus* and Axolotl is the same as that between *Xenopus* and mouse or human (Axolotl has fewer a.a. differences with mammalian species than with *Xenopus*). Vertebrate Msi-1 is very highly conserved in general at the amino acid level.

#### Msi-2 Isoforms

**FIGURE 3:**
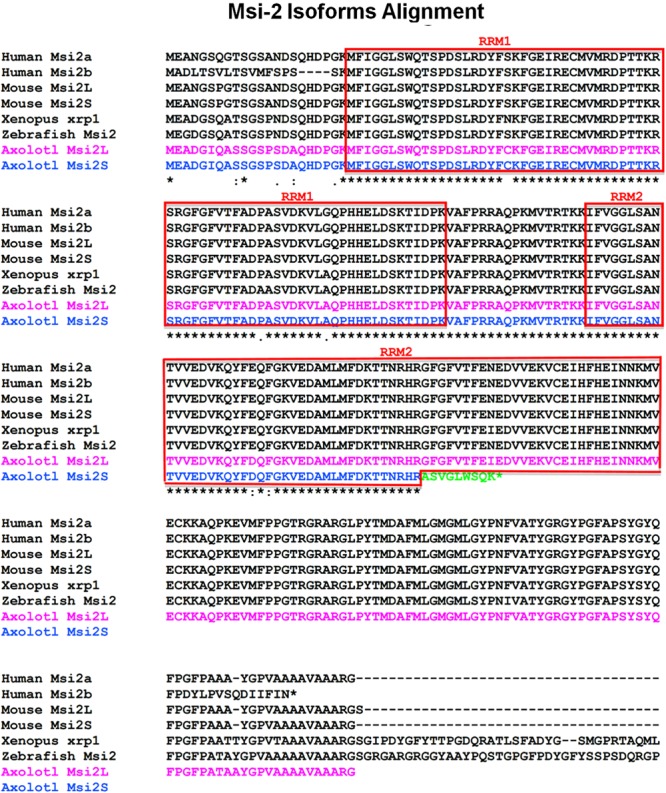
Msi-2 multispecies alignment, deduced amino acid sequences including *Xenopus* and Axolotl results. The RNA recognition motifs (RRMs) are indicated by the boxed areas labeled RRM1 or RRM2. Subcloning of Axolotl Msi-2 transcripts showed the presence of a shortened isoform ending with a truncated RRM2 as well as the long and short *Xenopus* Msi-2 isoforms described in **Figure 4**. For *Xenopus*, the original Msi-2 sequence is labeled xrp-1 as originally published. The C-terminal end is not shown for some species.

**FIGURE 4:**
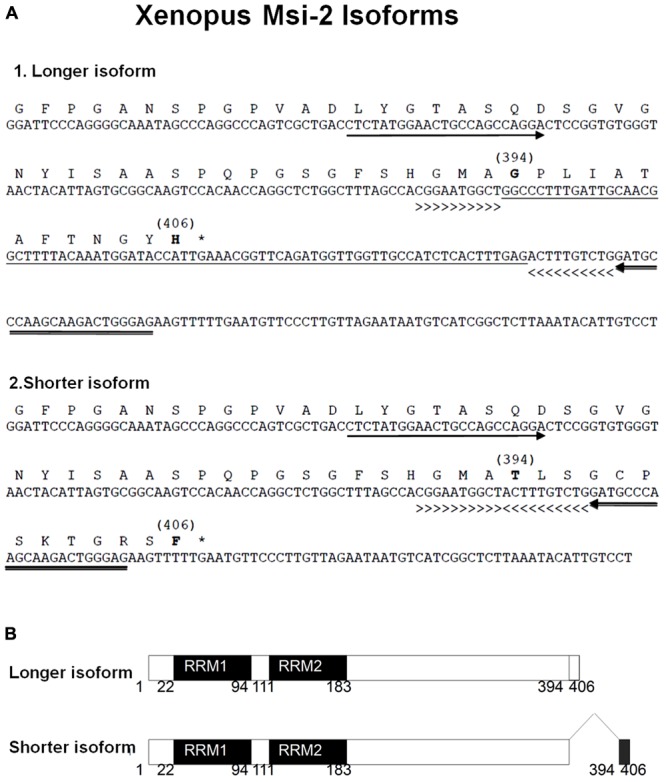
**(A)** The nucleotide and deduced amino acid sequences are shown for segments of two isoforms of *Xenopus* Msi-2 detected by RT-PCR. These isoforms were detected using one set of forward and reverse primers. The arrows under the nucleotide sequence show the position of forward (5′-CTCTATGGAACTGCCAGCCAGGA-3′) and reverse (5′-GATGCCCAAGCAAGACTGGGAG-3′) primers sequences. The longer isoform **(A1)** yielded a 203 bp band, while the shorter isoform band **(A2)** had 145 bp. The splicing insert (underlined DNA sequence) in the long isoform **(A1)** occurred between the greater-than (>>>>>) and the less-than signs (<<<<<). In the shorter isoform **(A2)**, the region underlined in **A1** was spliced out and the regions designated between the greater-than the less-than signs, respectively, were directly joined. The bracketed numbers above some amino acids indicate the differing region, and correspond to schematic **(A)**. The asterisks in **A1** and **A2** show the terminal codons. **(B)** The schematic for the deduced amino acid sequence region illustrates 100% homology from amino acid 1 to 393, between the longer and shorter band isoforms, with the alternatively spliced region from 394 to 406. The full length amino acid sequence of both isoforms has the same 406 amino acid length. RRM1 and RRM2 indicate, respectively, RNA-recognition motif-1 and -2.

Both *Xenopus* and Axolotl produce long and short transcripts of Msi-2 (**Figures 3, 4**). Cloning and sequencing of the *Xenopus* Msi-2 transcripts showed the existence of a lower molecular weight, alternatively spliced, form (**Figure 4**) in addition to the longer form reported by Good et al. (1993) as xrp-1. Both *Xenopus* Msi-2 isoforms contained complete mRNA-binding motifs. The deduced amino acid sequences in **Figure 4** show the region containing the splice (**Figures 2, 4A1**) and the schematic (**Figure 4B**) illustrates the proximity of the splice to the carboxy terminus.

As in *Xenopus*, a short isoform of Msi-2 was found in the Axolotl. However, the Axolotl short form contained a truncation of RRM2 that could affect mRNA-binding. The comparative amino acid alignment is shown in **Figure 3**. The truncation ended with a different 9 amino acid segment than is found in the Axolotl long form and in other species: ASVGLWSQK instead of GFGFVTFEI. Five of the 9 amino acids were non-conservative substitutions (Henikoff and Henikoff, 1992). Twenty amino acids of the Axolotl Msi-2 RRM2 were missing and the transcript was truncated at the end of the substitution. Both isoforms were included in the RT-PCR Axolotl Msi-2 band shown in **Figure 6** and were only detected by subcloning. Msi-2 expression in intact and regenerating adult cord is relatively weak (**Figure 6**).

### Semi-Quantitative RT-PCR

#### *Xenopus* RT-PCR Results

**FIGURE 5:**
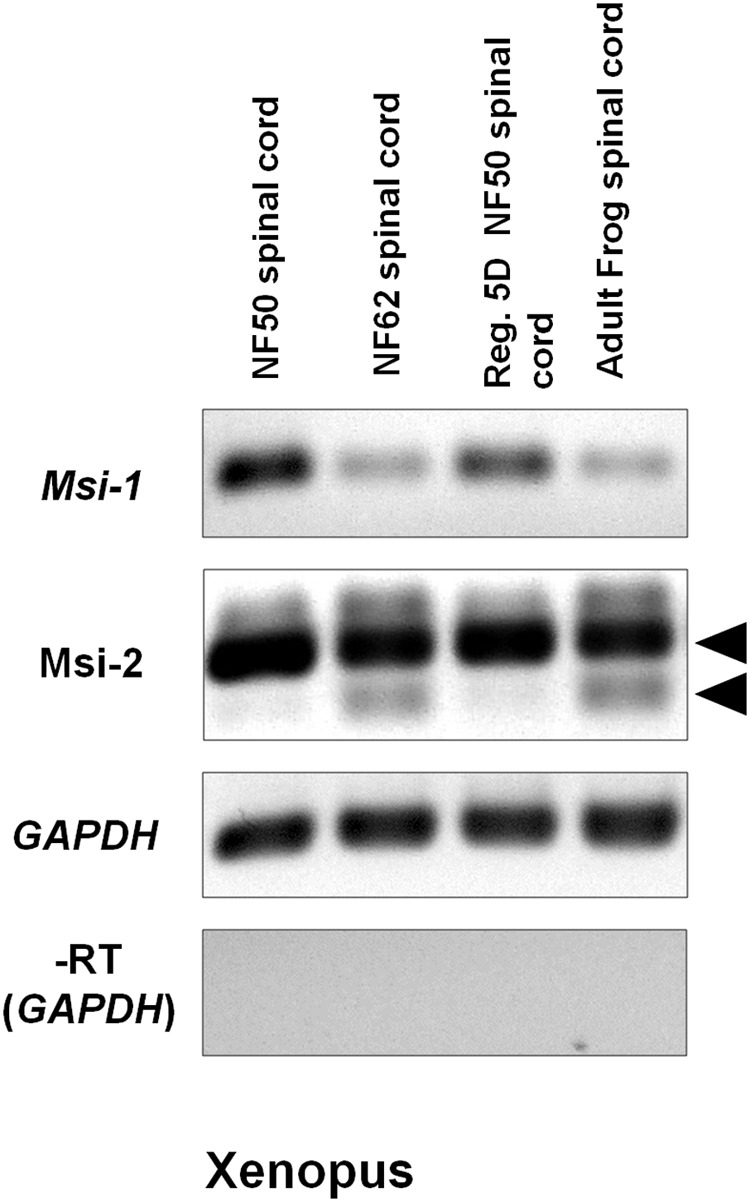
*Xenopus* Msi-1 and Msi-2 semi-quantitative RT-PCR gels show strong expression of Msi-1 in intact, regeneration-competent NF 50 spinal cord (gel column 1), and 5-day regenerating NF 50 spinal cord (Reg. 5D NF 50 spinal cord, gel column 3). Both Msi-1 and Msi-2 were reduced from NF 50 levels in intact NF 62 (non-regeneration-competent) and adult spinal cord (compare gel columns 1, 2). Msi-2 specific primers showed bands representing the two isoforms shown in the **Figure 4** alignments (arrowheads). The larger isoform was universally present. The smaller isoform (lower arrowhead) was present only in NF 62 and adult frog (compare gel columns 2, 4). GAPDH (glyceraldehyde-3-phosphate dehydrogenase) was used as the housekeeping control gene. The No-RT control (–RT) was performed without reverse transcriptase using the GAPDH primers. Each gene is shown in a separate row. All bands in each row are from one original gel. Negative was shown as for image clarity.

Semi-quantitative RT-PCR gel results in **Figure 5** showed a reduction in Msi-1 between the regeneration competent and incompetent stages in *Xenopus*. *Xenopus* Msi-1 was strongly expressed in spinal cord at NF 50–54, as described by Richter et al. (1990) and Good et al. (1993). Msi1 was expressed in the regenerating spinal cord after transection at NF 50, but did not show an increase between the intact (NF 50) and regenerating (5 days post-transected) spinal cord (**Figure 5**). Msi-1 expression was greatly reduced in NF 62 tadpoles (just prior to tail resorption) and adult frog spinal cord had equally weak levels of expression of Msi-1 (**Figure 5**).

On the other hand, *Xenopus* Msi-2 showed a different RT-PCR pattern. Msi-2 presented as two alternatively spliced transcripts. The larger isoform (**Figure 5** upper arrowhead) was equally expressed in NF 52, NF 62 and adult *Xenopus* spinal cord. The smaller isoform (**Figure 5** lower arrowhead) appeared only in non-regenerating NF 62 tadpoles and adult frogs.

#### Axolotl RT-PCR Results

**FIGURE 6:**
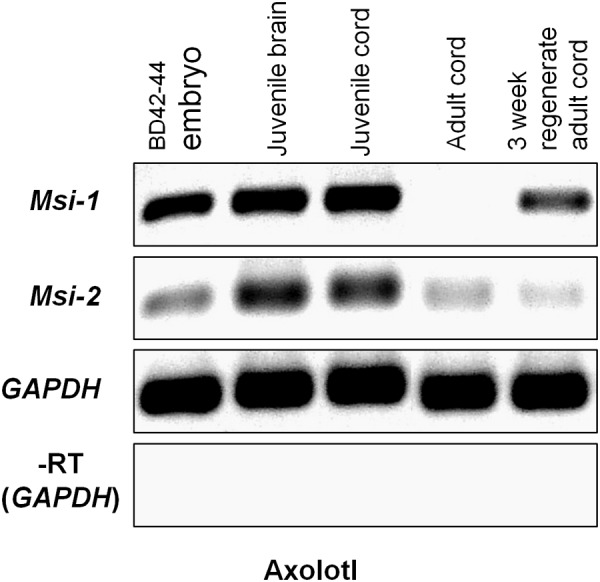
Axolotl Msi semi-quantitative RT-PCR gel. Msi-1 was strongly expressed in the pre-hatching/late tailbud stage embryo (BD 42–44; column 1), juvenile brain and spinal cord (gel columns 2, 3), but was absent in intact adult spinal cord (column 4). At 3 weeks post-transection in the adult (3 weeks regenerating adult cord) Msi-1was up-regulated (column 5). Msi-2 expression was stronger in the juvenile brain and spinal cord (columns 2, 3) than in the BD 42–44 embryo (column 1). Msi-2 was still expressed in intact adult spinal cord (column 4), and not up-regulated in regenerating adult cord (column 5). GAPDH was used as the internal housekeeping gene loading control, and (–RT) was performed without reverse transcriptase using the GAPDH primers. Each gene is shown in a separate row. All bands in each row are from one original gel (see Supplementary Figure 2) with limb regeneration sample bands excised here. Negative was shown as for image clarity.

Semi-quantitative RT-PCR was performed across unique nucleotide sequence zones outside of the Axolotl Msi mRNA-binding domains to avoid cross-reaction between Msi-1 and Msi-2 (**Figure 6**). Msi-1 was strongly expressed in pre-hatching/late tailbud stage embryos BD 42–44 (Bordzilovskaya and Dettlaff Axolotl staging series; Bordzilovskaya et al., 1989) and in juvenile Axolotl brain and spinal cord. Embryonic expression was weaker than in the juvenile CNS tissue, perhaps due to inclusion of some surrounding non-CNS tissue in the dissection while juvenile and adult cord are more easily removed from fully formed spinal column. Msi-1 expression in adult spinal cord (27–29 cm animals, 2+ years old) was faint. Regenerating spinal cord Msi-1 transcript levels were greatly increased compared with intact adult spinal cord. Unlike Msi-1, Msi-2 was still expressed in intact adult Axolotl spinal cord, but did not increase in the regenerating adult spinal cord.

### Tissue Localization

#### *Xenopus* Musashi

**FIGURE 7:**
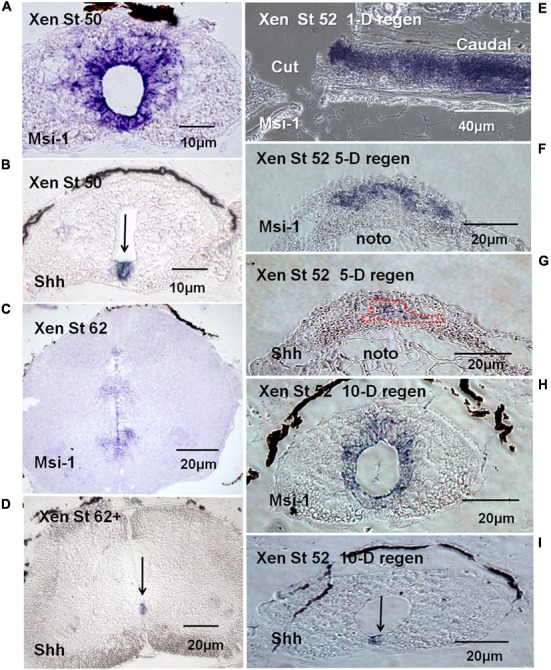
mRNA tissue distribution of *Xenopus* Msi-1 in developing and regenerating spinal cord using section *in situ* hybridization (blue reaction product). **(A)** Cross section of intact NF 50 spinal cord. Msi-1 was strongly expressed in the ependymal layer. **(B)** Shh mRNA localization in intact NF 50 spinal cord in the section adjacent to the one shown in “**A**.” Shh is expressed in the floorplate (arrow). **(C)** NF 62 spinal cord cross section showed Msi-1 was still expressed in the ependymal layer, but mostly ventrally located and weaker. **(D)** NF 62+ Shh mRNA is still expressed in the ventral floorplate, overlapping the Msi-1 expression (arrow). **(E)** Parasagittal section of NF 52 spinal cord 1-day (1-D) post-transection showed high level of Msi-1 expression throughout the cord, lesion site and caudal stump. **(F)** Cross section of NF 52 regenerating cord 5-days (5-D) post-transection showed a broad ventral arch of Msi-1 expression in the ependymal outgrowth. The dorsal portion of the notochord is visible below the outgrowth (noto). **(G)** Cross section of NF 52 regenerating cord 5-days (5-D) post-transection in the section adjacent to the one shown in “**F**”. Shh localization is largely in a complementary zone of outgrowth ventral to Msi-1 expression. (Combined Msi-1 and Shh expression shown in Supplementary Figure 6.) The dorsal portion of the notochord is visible below the outgrowth (noto). **(H)** Cross section of lesion site in NF 52 spinal cord 10-days (10-D) post-transection showed the reconstructed central canal with ependymal Msi-1 expression. **(I)** NF 52 spinal cord 10-days post-transection (10-D) in the section adjacent to the one in “**H**” showed Shh expression restored to floorplate location. Differential interference contrast optics, transmitted light images.

Section *in situ* hybridization showed that *Xenopus* Msi-1 mRNA was strongly expressed in the ependymal zone around the canal in NF 50 spinal cord (**Figure 7A**), including the floorplate. Floorplate Msi-1 was coexpressed with the DV axis patterning gene Shh (**Figures 7A,B**). Msi-1 expression also extends in a short plume dorsally from the central canal (**Figure 7A**). At the non-regenerating stage, NF 62+, Msi-1 was still expressed in the ependymal layer, but in localized zones of cells around the central canal (**Figure 7C**). The impression of reduced levels of Msi-1 at NF 62 in the *in situ* localization was supported by the semi-quantitative RT-PCR results (**Figure 5**).

Msi-1 was expressed in regenerating spinal cord tissue transected at NF 50–53. One day after lumbar cord transection at NF 52, Msi-1 was very strongly expressed in the cord, shown in parasagittal section (**Figure 7E**). Five days after lumbar transection, there was bulb-like outgrowth of tissue from the cranial and caudal stumps in the lesion site, and Msi-1 transcript existed in the dorsal and lateral parts of the regenerating tissue (**Figure 7F**). The 5 days regenerated Msi-1 expression formed an arched pattern in the outgrowth. *In situ* hybridization for Shh at 5 days of regeneration (**Figure 7G**), in the section adjacent to the one in **Figure 7F**, showed that the zone of Shh expression was roughly complementary to the Msi-1, found below the arch of Msi-1 expression (**Figure 7G**). By 10 days of regeneration, the cranial and caudal outgrowth had fused within the lesion site and reconstructed the central canal (**Figure 7H**). **Figures 7H,I** showed the restoration of normal coexpression of Shh and Msi-1 in the floorplate.

#### Axolotl Musashi

**FIGURE 8:**
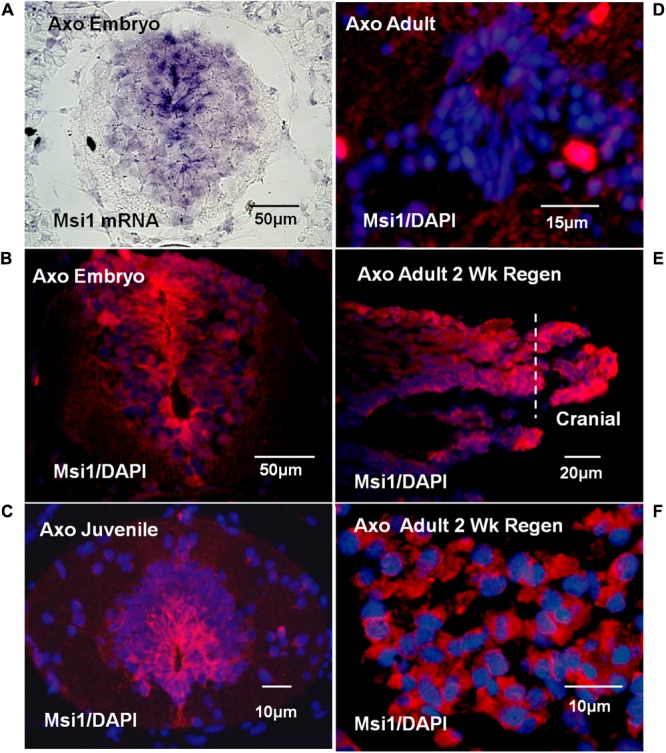
Axolotl mRNA (purple) or Msi antibody (red) tissue localization and DAPI nuclear label (blue). **(A)** Late tailbud embryo (BD 42–44) cross-section *in situ* hybridization for Msi-1 mRNA showed localization in ependymal region around the central canal and in gray matter. Differential interference contrast image. **(B)** Antibody localization in late tailbud embryo sections showed Msi-1 protein was present in the same regions as the mRNA shown in “**A**.” **(C)** Juvenile cord maintained Msi-1 expression in the ependymal zone, extending dorsally. **(D)** Intact adult Axolotl cord showed no Msi-1 protein expression, only DAPI-stained nuclei (blue). **(E)** Parasagittal section of 2 weeks adult regenerative outgrowth showed strong upregulation of ependymal Msi-1expression. **(F)** In cross section, at higher magnification than in “**E**” the mesenchymal nature of the Msi-1-positive 2-week adult regenerated outgrowth is shown. Fluorescence microscope images. All images are dorsal side up.

Tissue localization of Msi-1 in the Axolotl was performed using antibodies because *in situ* hybridization in adult urodele tissue produced unsatisfactory, intense background when compared with embryonic or larval tissue. The distribution of Msi-1 in the Axolotl was described in preliminary form in Sarria et al. (2008, published abstract). Axolotl Msi-1 protein expression changed during the progression from embryonic to juvenile to adult cord. In the late tailbud embryo (BD 42–44) both the radial glial/ependymal zone (around the central canal) and entire gray matter was positive at the mRNA and protein level (**Figures 8A,B**). The ependymal layer of juvenile spinal cord (10–15 cm, ∼6 months of age) showed strong localization extending dorsally and ventrolaterally into the gray matter (**Figure 8C**). In adult/sexually mature animals (>23 cm in length, 2+ years old) there was no detectable Msi-1 expression in the ependymal cells of the intact spinal cord (**Figure 8D**), only DAPI-labeled nuclei are present. Extensive Msi-1 expression in the mesenchymal ependymal outgrowth was seen in parasagittal and cross section following transection of adult Axolotl cord (**Figures 8E,F**).

#### Axolotl Shh Expression

**FIGURE 9:**
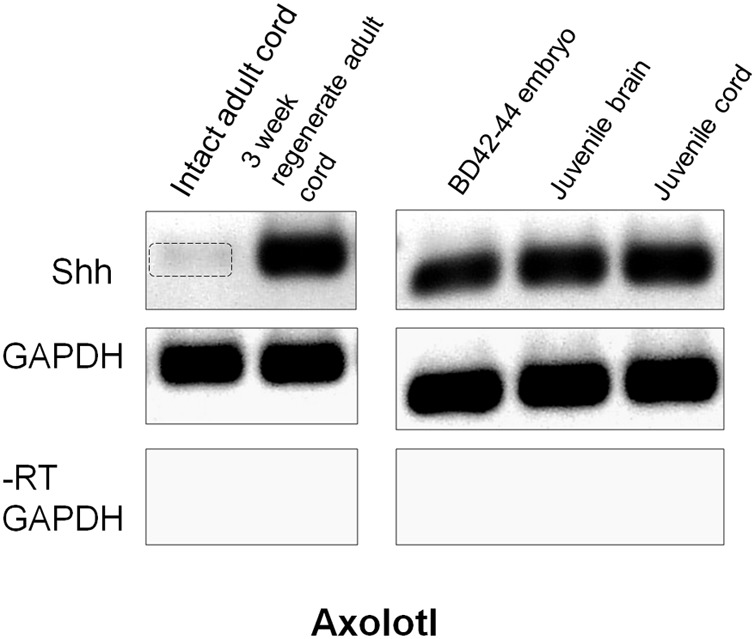
Axolotl Shh semi-quantitative RT-PCR. Intact adult Axolotl cord showed low levels of Shh (gel column 1). Expression was upregulated at 3 weeks regeneration (column 2). Intact late tailbud (BD 42–44) Axolotl embryo cord, juvenile cord and brain all showed high levels of Shh expression (columns 3–5). GAPDH was used as the internal housekeeping gene control, and the No-RT (–RT) was performed without reverse transcriptase using the GAPDH primers. Each gene is shown in a separate row. All bands in each row are from one gel, but limb regeneration results were excised and only CNS results that flanked limb samples are shown (see original rows in Supplementary Figure 3). Negative was shown as for image clarity.

Examination of expression of the ventral patterning gene Shh was performed using RT-PCR because of unsatisfactory background in adult Axolotl cord *in situ* hybridization and batch inconsistency of Shh antibodies. Semi-quantitative RT-PCR showed Shh to be present at very low levels in intact adult Axolotl cord and strongly upregulated at 3 weeks of regeneration (**Figure 9**). In contrast, late tailbud embryos, juvenile spinal cord and juvenile brain Shh expression was uniformly high (**Figure 9**). The upregulation of Shh during regeneration was similar to the intact/regenerating adult tissue pattern seen for Msi-1 expression (**Figure 6**).

#### GFAP Expression

**FIGURE 10:**
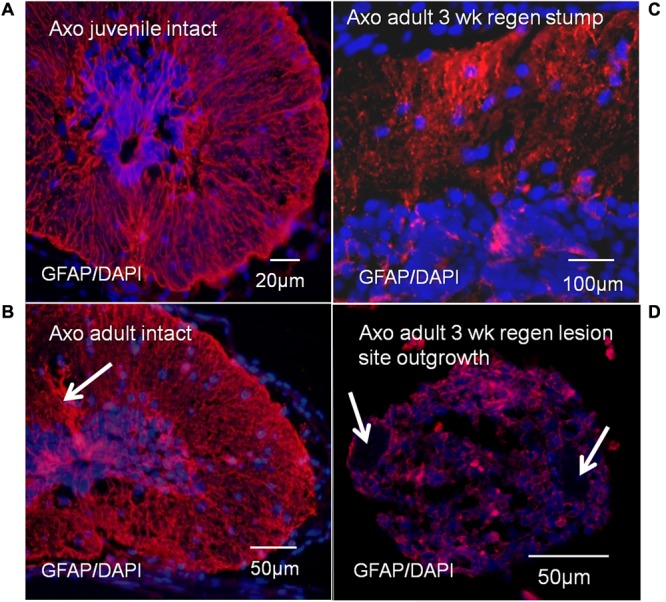
Axolotl GFAP antibody labeling (red), with DAPI labeled nuclei (blue). GFAP labeling showed bundles of radial ependymal fibers in sections of the intact juvenile **(A)** and adult **(B)** Axolotl spinal cord. Arrow in **B** indicates the bundle of tanycyte radial fibers extending dorsally. There was also cytoplasmic GFAP content at both stages. In the stump region at 3 weeks of regeneration, GFAP was reduced in the radial processes **(C)** and was at low levels in the regenerative outgrowth **(D)**. Arrows indicate location of the denticulate ligaments associated with meninges surrounding the regenerative outgrowth **(D)**. The bright red spots are red blood cell autofluorescence. Fluorescence microscope images.

Antibody localization was performed for GFAP, the predominant intermediate filament protein of Axolotl radial ependymal processes (**Figure 10**). These results provide tissue organization context for the Msi-1 antibody images of juvenile vs. adult cord and adult regeneration stages. GFAP antibody labeling shows bundles of radial ependymal fibers in sections of the intact juvenile (**Figure 10A**) and adult (**Figure 10B**) Axolotl spinal cord. The GFAP extends into the “cell body” closer to the central canal in both stages. In the stump region, at 3 weeks of regeneration, GFAP is reduced in the periphery (**Figure 10C**), during retraction of radial processes. There were low levels of GFAP in the 3 weeks regenerative outgrowth (**Figure 10D**), reflecting the gradual breakdown of GFAP during mesenchymal ependymal outgrowth (O’Hara et al., 1992).

#### Proliferation

**FIGURE 11:**
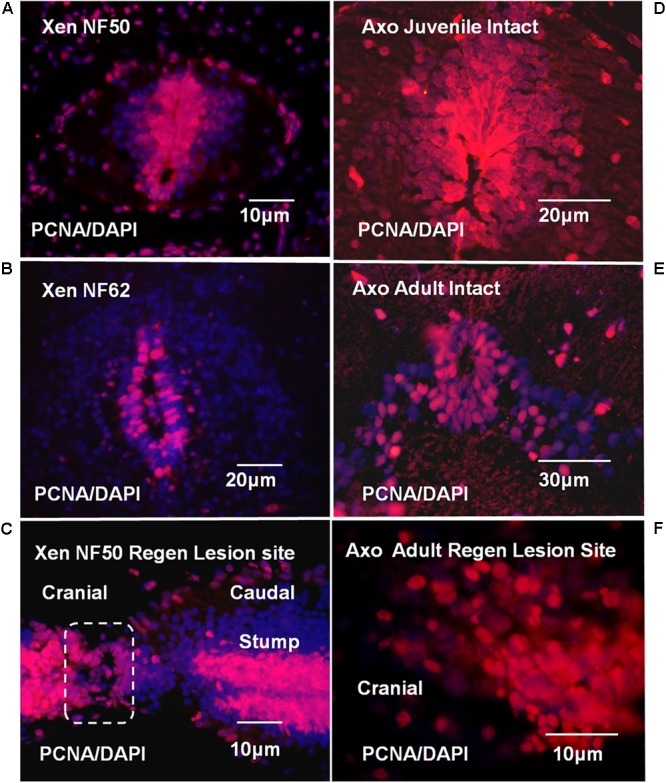
*Xenopus* and Axolotl cell proliferation; PCNA antibody (red) and DAPI nuclear stain (blue) are used throughout. **(A)** PCNA staining in NF 50 spinal cord. The ependymal layer cells are PCNA-positive. The PCNA labeled region coincides with the region of Msi-1-positive cells shown in **Figure 7A**. **(B)** NF 62 spinal cord with PCNA antibody and DAPI staining. Some cells in the ependymal layer are still proliferating, but in a reduced area around the central canal. **(C)** Parasagittal section shows regenerative outgrowth 7-days (7D) post-transection regenerating NF 50 tadpole spinal cord. The zone of outgrowth and lesion site is indicated within the dashed line. The entire ependymal population contains proliferating cells. **(D)** Intact juvenile Axolotl (∼12 cm; less than 6 months old) spinal cord cross section shows extensive ependymal and gray matter PCNA labeling. There is a substantial dorsal extension of labeled ependymal cells. **(E)** Intact adult Axolotl (∼25 cm; approximately 2–3 years old) spinal cord cross section shows strong, but reduced PCNA labeling in the ependymal zone. The dorsal plume of PCNA-labeled cells no longer exists and labeling is reduced in the gray matter laterally. **(F)** Parasagittal section showing PCNA-positive cells in mesenchymal ependymal outgrowth. (Supplementary Figure 8 shows DIC image of the section with the outgrowth and stump region labeling for orientation.) Fluorescence microscope images.

The localization of proliferative ependymal cells at NF 50 was coincident with the Msi-1 distribution (**Figures 7A, 11A**). There was a dorsal plume of proliferating cells in addition to proliferating cells surrounding the central canal. By NF 62, the zone of proliferative cells was reduced, consistent with slower but continuing tadpole growth. The 7 days regenerating NF 50 tissue was densely PCNA-labeled in both cell outgrowth and adjacent stump tissue (**Figure 11C**). The zone of proliferation overlapped the area of Msi-1 labeling (**Figure 7A**). The change in pattern of proliferation in *Xenopus* could be related to a progressive decrease in the rate of growth of the tadpole and may not be directly related to the loss of regenerative capacity.

The proliferative pattern changed extensively between juvenile and adult Axolotl cord. Localization of PCNA-positive proliferating cells in juvenile Axolotl cord (**Figure 11D**) overlapped the region of Msi-1 expression (**Figure 8C**), but gray matter cell proliferation was also present. In the adult spinal cord, PCNA-positive cells were found around the central canal and adjacent gray matter (**Figure 11E**). Msi-1 was not detected in the adult ependymal zone, though those cells were PCNA-positive (**Figure 8D**). In the adult Axolotl cord, the region of proliferative cells was much more restricted than in the juvenile. Within the lesion site PCNA-positive cells were growing out into the lesion site (**Figure 11F**).

### Cell Culture Behavior

#### EGF Effects

**FIGURE 12:**
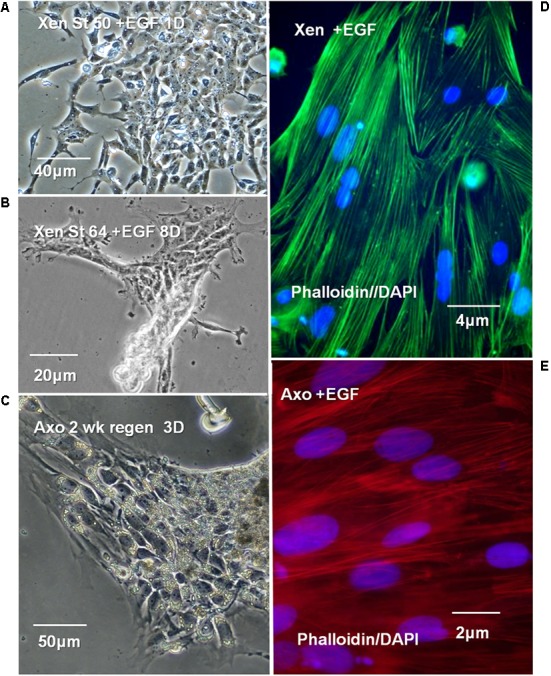
*Xenopus*, Axolotl cell culture in EGF-containing culture medium (+EGF). (A–C) Phase contrast images. **(A)** Control NF 50 *Xenopus* ependymal cells in +EGF, 1 day (1D) 6 to 8 large patches of cells adhere to each dish. **(B)** NF 64 cultures: small, islands of cells, 4–6/dish, exhibit poor attachment and growth even at 8 days (8D) in culture. **(C)** Control adult Axolotl 2 weeks regenerating ependymal outgrowth explants in EGF at 3 days (3D) culture, patches of cells continue to expand in culture. **(D,E)** Fluorescent-Phalloidin probes are used to show the organization of F-actin in *Xenopus* and Axolotl +EGF cultures. **(D)** Alexa fluor 488-Phalloidin green fluorescence in a +EGF *Xenopus* ependymal culture shows typical mesenchymal cell F-actin organization, parallel to the long axis. **(E)** Rhodamine-Phalloidin red fluorescence in a +EGF Axolotl culture shows cell F-actin organization typical of mesenchymal outgrowth. DAPI nuclear stain used in “**D,E**” (blue). **(D,E)** Fluorescence microscope images.

NF 50 *Xenopus* ependymal cultures formed 6–8 large mesenchymal patches of cells on fibronectin-coated dishes in EGF-containing medium (**Figure 12A**, 1-day *in vitro*). Under the same culture conditions NF 64 (non-regeneration-competent) ependymal cells formed 4–6, small patches that did not expand even with extended times in culture (**Figure 12B**, 8 days *in vitro*). Axolotl ependymal explants attached and expanded rapidly in culture (**Figure 12C**), described in detail in previous publications (Chernoff et al., 1990, 1998; O’Hara and Chernoff, 1994). Fluorescent–phalloidin probes were used to visualize the ependymal cell filamentous actin (F-actin) distribution in EGF-containing *Xenopus* and Axolotl cultures to confirm the form of the cell outgrowth. The F-actin organization confirmed that both NF 50 *Xenopus* and Axolotl ependymal growth in EGF-containing medium to be mesenchymal (**Figures 12D,E**). The adhesion of the NF 64 cultures was weak: cells did not remain substratum-attached during processing for F-actin staining.

#### Msi-1 and PCNA *in Vitro*

**FIGURE 13:**
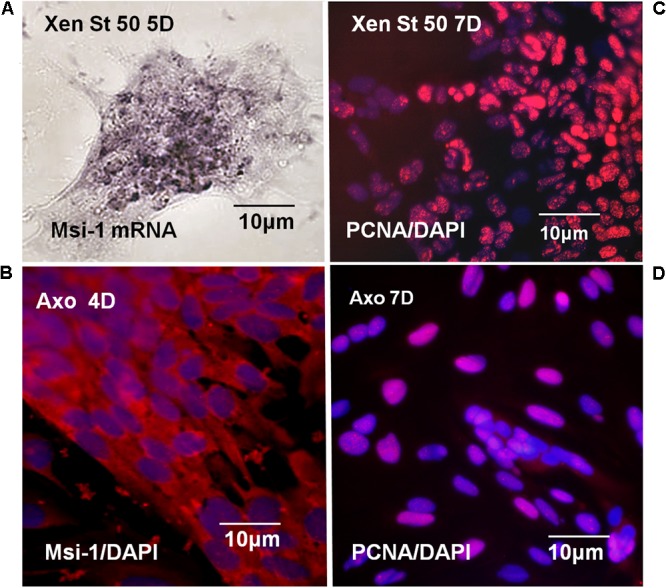
*Xenopus* and Axolotl Msi-1, proliferation *in vitro*. **(A)** NF 50 *Xenopus* dish *in situ* hybridization with Msi-1 riboprobe shows Msi-1 expression is maintained in culture. Bright field image **(B)** Axolotl anti-Msi-1 antibody localization in 2 weeks regenerating mesenchymal ependymal outgrowth. Msi-1 expression is maintained in culture. **(C)** Labeling with PCNA antibody showed NF 50 *Xenopus* ependymal cells are proliferative in culture. **(D)** Labeling with PCNA antibody showed 2 weeks regenerating adult Axolotl mesenchymal ependymal outgrowth is proliferative. **(B–D)** Fluorescence microscope images. All of the cultures are in EGF-containing medium.

Msi-1 expression and proliferative capacity of *Xenopus* and Axolotl ependymal cells was compared in culture. Both regeneration-competent *Xenopus* and regenerating adult Axolotl ependymal cells maintained Msi-1 expression *in vitro*, shown at the mRNA and protein levels, respectively (**Figures 13A,B**). PCNA antibody labeling showed that both regeneration-competent *Xenopus* and Axolotl ependymal cultures also continued to proliferate in culture (**Figures 13C,D**). Cultures from NF 64 tadpoles produced small patches of cells (**Figure 12B**) that did not remain attached through the PCNA antibody labeling procedure, suggesting weak adhesion. These results link studies of *in vivo* and *in vitro* ependymal cell behavior. So, the ependymal culture system was used to test the growth modulators that support mesenchymal outgrowth (EGF) and to determine if regeneration-competent cells from amphibians with different modes of maturation, at different life stages produce similar responses to EGF as a tissue organization regulator. Maintenance of Msi-1 during mesenchymal outgrowth in the presence of EGF could be correlated with Notch signaling-related plasticity of the cells in the regeneration process *in vivo* and *in vitro*.

## Discussion

### Why Musashi?

Neural stem cell properties are important in regeneration (rev. Becker and Becker, 2015). The stem cell-related genes Sox2/3 (*Xenopus*) and Sox2 (Axolotl) are key mediators of neural stem cell properties in amphibian larval stages in both tail and body region cord (Gaete et al., 2012; Fei et al., 2014; Muñoz et al., 2015). Sox2 is downregulated in NF 66, non-regenerating, *Xenopus* (Gaete et al., 2012). Why examine Musashi, then? Conditional knock-out of RBPj (Recombination Signal Binding Protein For Immunoglobulin Kappa J Region, also known as RBPjkappa), which forms a transcription factor heterodimer with the processed Notch intracellular domain, leads to depletion of Sox2 in mammalian neural stem cells in the brain subventricular zone. This suggests that Notch signaling could act upstream of Sox2 in neural stem cell behavior (Ehm et al., 2010). Notch signaling regulates neural stem cell numbers and prevents premature onset of neuronal differentiation (Androutsellis-Theotokis et al., 2006). Msi-1 maintains Notch signaling, so Msi is an important element of stem cell maintenance (Imai et al., 2001; Ehm et al., 2010; Gaete et al., 2012; Fei et al., 2014; Muñoz et al., 2015). The connections among different stem cell maintenance signaling pathways and the relationship of stem cell maintenance to regeneration are not completely understood, so the roles of all of the important pathways and pathway members need to be examined.

The action of Msi in maintenance of Notch signaling is a well-described phenomenon (rev. Imai et al., 2001; Horisawa et al., 2010). There is also literature on Msi genes and regeneration (Richter et al., 1990; Sakakibara et al., 1996; Kaneko et al., 2000; Imai et al., 2001; Sakakibara et al., 2001; Faigle and Song, 2013). Musashi is likely to be involved specifically in the ability of amphibian ependymal cells to engage in spinal cord regeneration (rev. Chernoff et al., 2002; Faigle and Song, 2013). In a comparative study of regenerating amphibians, such as this one, Msi-1 is a good marker of the regenerative process.

Msi also plays a role in maintenance of cell cycling through a competitive interaction with the mRNA-binding protein HuC/D or HuD (depending on species). Msi-1 inhibits the cell cycle regulator p21, maintaining cell cycling (Battelli et al., 2006). The relationship between Msi-1 and the Hu mRNA binding proteins is complicated, because HuD can also stabilize Msi mRNA and prolong its action (Ratti et al., 2006). The levels of Msi-1 were seen to decline in *Xenopus* between the regeneration competent and non-competent stages (**Figures 5, 7**). In a Supplementary Figure 7, a *Xenopus* HuD analog can be seen in the hindbrain ependymal zone. The relationship there between the two mRNA-binding proteins could be either supportive or competitive. Differential effects of Msi-1 on Notch-mediated stem cell properties and p21-mediated cell cycling were not addressed in this study.

### Changes with Stage and State in *Xenopus* and the Axolotl

**Table 1** shows a summary of the expression changes of the markers examined in this paper.

**Table 1:** Expression summary for markers, species, stages of development, and intact vs. transected cord.

Species and Stage	Msi-1 mRNA, Ab	Msi-2L	Msi-2S	Shh mRNA	GFAP Ab	PCNA Ab
		mRNA	mRNA	or Ab		
*Xenopus* NF 50–53	Strong, ependymal, gray matter	Strong	Absent	Strong	No GFAP gene	Strong ependymal
*Xenopus* NF 50–53 regeneration	Upregulated	Strong	Absent	Changed distribution	–	Strong ependymal
*Xenopus* NF 62	Low; restricted ependymal zones	Strong	Strong	Present	–	More restricted than NF 50
*Xenopus* NF 62+ cut	(Suppl) No increase over NF 62+ intact				–	
*Xenopus* adult	Lower	Strong			–	
Axolotl embryo	Strong; ependymal, gray matter	Medium		Strong		
Axolotl juvenile	Strong; ependymal, gray matter	Strong	Present	Strong	Radial processes	Ependymal, gray matter
Axolotl adult	Absent	Low	Present	Low	Radial processes	Ependymal, gray matter, less extensive than juvenile
Axolotl adult regeneration	Strongly upregulated	Low	Present	Strong	Reduced	Ependymal outgrowth and stump

*mRNA or antibody expression is listed according to species, life stage and intact or lesioned cord for Msi1, long and short forms of Msi-2 (Msi-2L, Msi-2S), sonic hedgehog (Shh), glial fibrillary acidic protein (GFAP) and proliferating cell nuclear antigen (PCNA) expression. (Suppl) Indicates data in the supplementary section from experiments that were performed in a related, but not strictly equivalent form to that included in the paper.*

At its simplest, using Msi-1 as a marker, the major difference reported here is between gene expression remaining active in an intact *Xenopus* tadpole spinal cord as opposed to gene expression requiring activation after adult Axolotl cord injury. Secondarily, Msi-2 plays a different role than Msi-1. Some form of Msi-2 is active in the amphibian CNS throughout life, but some isoforms, such as the lower molecular weight form of *Xenopus* Msi-2 described here, may be a marker for loss of regeneration competence.

### Comparing *Xenopus* and Axolotl Regeneration

There is a fundamental difference in CNS regenerative capacity between anurans and urodeles. Both require the expression of neural stem cell gene expression pathways, but in anurans the stages at which CNS regeneration is successful (pre-metamorphosis) ends well before the animal reached adulthood.

Many studies have used either anuran (frog and toad) or urodele (salamander and newt) amphibians as a spinal cord regeneration model. There are changes in neural stem cell behavior for both anurans and urodeles in the course of growth and differentiation of the CNS. There are, for example, known qualitative differences in the formation of new neurons among different life stages in anuran and urodele amphibians (Detwiler, 1947; Holtzer, 1951, 1952; Butler and Ward, 1965, 1967; Stensaas, 1983; Davis et al., 1989).

The Clawed Frog *Xenopus laevis* and the Axolotl *Ambystoma mexicanum* are widely used examples of those two orders of Amphibia. Each species has advantages and disadvantages in regeneration studies.

*Xenopus* has proven amenable to molecular biology studies. However, the *Xenopus* regeneration competence period is only pre-metamorphosis (rev. Shi, 2000). As thyroid hormone levels rise, peak and fall during amphibian metamorphosis, gene expression is greatly altered (Veldhoen et al., 2002).

Axolotl regeneration is not complicated by metamorphosis. However, molecular biology studies in the Axolotl have lagged behind *Xenopus* because of practical considerations: longer development and generation times and the presence of a very large genome. The molecular biology of the Axolotl is improving, however (Smith et al., 2005; Monaghan et al., 2007; Keinath et al., 2015). The Axolotl genome has just been sequenced and assembled (Nowoshilow et al., 2018). While the Axolotl regenerates throughout its life, there are changes in the speed of regeneration, cell behavior in intact vs. regenerating tissue, and gene expression patterns among larvae, juveniles and adults (rev. Chernoff et al., 2003). Larval regeneration is more rapid than juvenile regeneration, which is more rapid than that in the adults (rev. Piatt, 1955).

The Axolotl is also neotenic; it remains gilled and aquatic as an adult, failing to go through most aspects of metamorphosis. For example, the parathyroid glands remain undeveloped like those in a larval amphibian, so its calcium regulation differs, it retains lateral line neurons and fails to develop a propulsive tongue for capturing prey (Clarke, 1983; Feder and Burggren, 1992); and continues growing throughout adult life. However, naturally metamorphosed salamanders including the newts, regenerate and experimentally metamorphosed Axolotls still regenerate CNS as adults (Chernoff et al., 2003; rev. Tanaka and Ferretti, 2009; Amamoto et al., 2016). CNS regeneration appears, to a significant extent, to be a fundamental urodele amphibian characteristic, not a result of neoteny. The Axolotl, like *Xenopus*, also breeds relatively easily in captivity so no animals need be collected from the wild.

Larval (tadpole) *Xenopus, Xenopus* froglets, and Axolotls at all life stages, are all animals with growing spinal cords in which the ependymal cells have a role in growth that may be separate from regeneration (Dent, 1962; Mitashov and Maliovanova, 1982; Chernoff et al., 2002, 2003; Becker and Becker, 2015). The changes that occur at different stages within an anuran or urodele species can be just as critical as anuran vs. urodele differences. Life stage-dependent differences described in this paper include changes in expression of the stem/progenitor cell markers Msi-1 and Msi-2, as well as proliferative behavior and growth factor responsiveness.

#### *Xenopus* Msi-1 *in Vivo*

The *Xenopus* Musashi-1 homolog was strongly expressed in the ventricular zone of spinal cord at NF 50 and remains strongly expressed during the regeneration process (**Figures 5, 7**). This is expected as Msi-1 expression was initially found in the ventricular zone in the developing *Xenopus* brain through NF 50 (Richter et al., 1990). During regeneration, Msi-1 was highly expressed in the cord stump tissue, as well as in the regenerative outgrowth (**Figures 7E,F**), consistent with maintenance of expression in intact cord at that stage. The Msi-1-positive cells were localized around the central canal, which coincides with the zone that contains neural stem cells in the mouse and the Sox2/3 positive zone in *Xenopus* (Sakakibara et al., 1996; Muñoz et al., 2015). The decline of Msi-1 expression in *Xenopus* at NF 62+ is consistent with the decline in the neural stem cell gene Sox2/3 (Gaete et al., 2012; Muñoz et al., 2015). The most prominent zone of Msi-1 expression at NF 62 by *in situ* hybridization (**Figure 7C**) was in the ventral zone associated with oligodendrocyte production (Maier and Miller, 1997), rather than being expressed strongly and evenly in all ependymal cells.

#### *Xenopus* Msi-2 *in Vivo*

There were two different size isoforms of the *Xenopus* Msi-2 homolog, and the presence of the shorter isoform correlated with loss of regeneration competence (**Figure 5**). Long and short isoforms contained both of the mRNA-binding domains (**Figure 4**), so the functional significance of the shift in abundance of these isoforms is not known. The existence of isoforms of *Xenopus* Msi-2 is consistent with observations in mouse, where Msi-1 and Msi-2 both have alternatively spliced isoforms (Sakakibara et al., 2001). Spatial distribution of Msi-2 is shown in Supplementary Figure 7, but the signal, which coincides with the dorsal region of Msi-1 expression, may be incomplete ventrally due to persistent erosion problems during the section *in situ* process. In mouse, Msi-2 has a same spatial expression pattern in spinal cord as Msi-1 (Sakakibara et al., 2001).

#### Axolotl Msi-1 *in Vivo*

Axolotl Msi-1 showed conspicuously different patterns of expression across developmental stages and between regenerating and intact adult spinal cord. In addition, the pattern differed completely from that seen in *Xenopus*. The presence or absence of Msi-1 expression may reflect the developmental state of the Axolotl CNS. Msi-1 levels were equal and strong in pre-hatching Axolotl embryos and in juvenile nervous system where growth is rapid and development continues (**Figures 6, 8**). The absence of Msi-1 in intact adult cord and reappearance after injury (**Figures 6, 8**) could be related to the relative stability of the cord in a 2- to 3-year-old animal.

#### Axolotl Msi-2 *in Vivo*

The difference seen between Msi-1 and Msi-2 across Axolotl life stages (**Figure 6**) suggests that Msi-2 may play a different role in Axolotl ependymal cells than it does in *Xenopus* or that there are additional, undetected, isoforms of Axolotl Msi-2. The high levels of Msi-2 seen across *Xenopus* stages are not present in the Axolotl: the adult Axolotl has low levels of Msi-2 that do not upregulate during regeneration.

Spatial localization studies for Axolotl Msi-2 failed: available antibodies produced non-specific staining. Also, *in situ* hybridization beyond the early larval stages is problematical for adult Axolotl and other urodele CNS tissue for unknown reasons (e.g., Zhang et al., 2000; Makanae et al., 2016). This is not meant as a criticism of the work from any laboratory: something in Axolotl tissue produces extraordinarily high background in adult urodele CNS so that only a very high signal shows over that background.

### Structural Similarities, Differences and Regeneration

#### Ependymal Zone Organization and Marker Expression

##### Xenopus

Dorsal expansion of the *Xenopus* ependymal zone starts soon after hatching (Roberts, 2000). The stage 50–52 *Xenopus* ependymal cells showed a dorsal/dorsolateral extension of Msi-1 expression (**Figure 7A**). In NF 62+ tadpoles the pattern was different with a reduced Msi-1 expression generally and isolated zones of stronger expression flanking the central canal (**Figure 7C**). The most ventral of these areas corresponds to a zone generating oligodendrocytes in the developing cord of other species (rev. Leigh and Maden, 2005).

##### Axolotl

In the Axolotl, the late tailbud stage embryo had broad expression of Msi-1 mRNA and protein throughout the spinal cord (**Figures 8A,B**) that would be expected in a vertebrate embryo. The intact juvenile Axolotl cord showed a dorsal plume of Msi-1 expression even more extensive than that in young *Xenopus* tadpoles (**Figure 7C**). The ependymal zone in the adult Axolotl cord has multiple ranks of cells (nuclear stain only **Figure 8D**) that indicate an absence of a pronounced dorsal plume of cells as well as an absence of Msi-1 expression.

The dorsal extension of the ependymal zone of the spinal cord may be the common characteristic of amphibian CNS development. In both *Xenopus* and the Axolotl the dorsal extension of ependymal cells exists well beyond the embryonic stages, into the juvenile stage in the Axolotl (**Figures 8C, 11D**). In embryos this is the region in which populations of dorsal interneurons are produced during spinal cord development (rev. Leigh and Maden, 2005). It is also known to be the zone in which the tanycytes, a specialized dorsal ependymal population will be present in the adult (**Figure 10B**; Bodega et al., 1994; Meletis et al., 2008). Pax7 and Pax6 protein are still present in larval and adult Axolotl cord, so neurogenesis from the dorsal plume ependymal cells is a possibility (Mchedlishvili et al., 2007). However, the formation of tanycytes, dorsal ependymal cells with close blood vessel associations that transfer material between the cerebrospinal fluid and blood, is a strong possibility at these late stages (rev. Moore, 2016). Formation of tanycytes would also require dorsal-ventral regionalization signals within the ependymal zone.

##### Radial processes, GFAP

There are similarities and differences in the structure of regeneration competent (NF 50–53), non-regenerating (NF 62+) *Xenopus* spinal cord and Axolotls. Both the neotenic adult Axolotl (**Figure 10B**) and metamorphosed urodeles, such as *Pleurodeles waltl* (the Spanish Ribbed Newt) continue to maintain radial ependymal fibers as adults that terminate in endfeet at the pial surface of the cord (rev. Tanaka and Ferretti, 2009). The radial processes are maintained in adult anuran cord, as well, (rev. Tanaka and Ferretti, 2009). As seen in *Xenopus*, retention of radial ependymal cell processes is not a guarantee of regeneration competence (rev. Chernoff et al., 2003).

Loss of GFAP content as ependymal cells reorganize to produce regenerative outgrowth is a common urodele feature. In *Pleurodeles waltl*, during tail bulb outgrowth, GFAP is downregulated and vimentin and nestin intermediate filament proteins are upregulated (Walder et al., 2003). Based on antibody localization, the literature has suggested for years that there are GFAP-containing intermediate filaments in the radial glial fibers in all amphibians studied, urodele or anuran, though the regional distribution of GFAP+ cell processes along the dorsal-ventral axis can vary by species (Bodega et al., 1994; rev. Tanaka and Ferretti, 2009). However, recent studies show that *Xenopus laevis* and *Xenopus tropicalis* do not have GFAP genes, and that GFAP antibodies are crossreacting with other intermediate filament proteins (Gervasi et al., 2000; Martinez-De Luna et al., 2017). The suggestion is that though the amphibian orders urodela (or caudata) and gymnophiona (caecilians) have a GFAP gene, the evolution of anura resulted in loss of the ancestral gene and they use vimentin or peripherin in its place (Gervasi et al., 2000; Martinez-De Luna et al., 2017). It is difficult to see how substitution of one intermediate filament protein for another would cause spinal cord regeneration failure, when there is so much intermediate filament heterogeneity in amphibians ependymal radial processes to start with Bodega et al. (1994). The radial process intermediate filament proteins do, however, represent a useful set of markers of ependymal cell reorganization during regeneration.

##### Ependymal reorganization and regeneration

The *in vitro* results presented here (**Figures 12, 13**) establish some equivalency between the *in vivo* and *in vitro* behavior of the cells that is useful in a discussion of tissue reorganization for regeneration. The disorganization of amphibian ependymal cells for regenerative outgrowth, and later reformation of a central canal are both critical to regeneration of a functional spinal cord.

The *in vivo* regenerative outgrowth process constitutes a loss, or partial loss, of epithelial organization. This is manifested as epithelial-mesenchymal transition (EMT) in the Axolotl body cord (**Figure 10**), and as bulb-like outgrowth in other urodeles such as *Pleurodeles waltl* and *Notophthalmus viridescens* (Eastern Red-Spotted Newt) (Walder et al., 2003; Zukor et al., 2011). The bulb-like outgrowth, with loss of basal lamina and a partial disorganization of the epithelial ependymal cells could represent a form of collective cell migration into the extracellular matrix-filled lesion site (Friedl and Gilmour, 2009).

##### EGF and ependymal cells

Epidermal growth factor, along with fibroblast growth factor-2 (FGF-2), can activate ependymal cell proliferation when infused after mammalian SCI (rat, Kojima and Tator, 2002). EGF is also one of several growth factors that can support epithelial-to-mesenchymal transition through apical transmembrane receptor tyrosine kinases (rev. Savagner, 2001). EGF is known to support cell migration modes associated with both edge cell-mediated collective migration or fully mesenchymal migration in EMT (Barrandon and Green, 1987; Wells et al., 1999; Savagner, 2001; Friedl and Gilmour, 2009; Park and Han, 2009). Present and prior studies show that EGF maintains Axolotl ependymal cells in a state of mesenchymal outgrowth *in vitro* for extended periods (**Figure 13**; O’Hara and Chernoff, 1994; Chernoff et al., 1998). Although the *Xenopus* NF 50–53 outgrowth *in vivo* is more bulb-like, with only partial loss of epithelial structure, the *Xenopus* ependymal cells responded to EGF *in vitro* with mesenchymal outgrowth like that of the Axolotl cells (**Figure 13**). This difference between *in vivo* and *in vitro* ependymal outgrowth in *Xenopus* could reflect both substrate and culture medium conditions that differ from the *in vivo* environment that induce changes in integrins and growth factor receptor production.

### Cell Proliferation and Regeneration

#### *Xenopus in Vivo* and *in Vitro*

In the rapidly growing/developing CNS of NF 50 *Xenopus* tadpoles even intact cord is Msi-1-positive and highly proliferative (**Figure 11A**). Ependymal outgrowth into the lesion site is also highly proliferative (PCNA+; **Figure 11**). Proliferation is maintained *in vitro* (**Figures 11A,C, 12A,C**). The PCNA+ status of the entire NF 50–53 *Xenopus* ependymal zone makes it likely that proliferation forms an important part of regenerative outgrowth from the stump in addition to any migratory cell behavior.

At NF 62, the zone of proliferating cells was reduced *in vivo* (**Figure 11B**). This could be related to a decrease in growth rate, or it may be related to the accompanying decrease in Msi-1 levels (**Figure 7C**) and a decrease in neural stem cell properties reported by others in NF 62+ spinal cord (Muñoz et al., 2015). In culture, the change in behavior at NF 62+ is manifested partly as poor adhesion, which made PCNA and Msi-1 antibody staining procedures impossible.

#### Axolotl *in Vivo* and *in Vitro*

Axolotl PCNA labeling showed that cell proliferation in the body region spinal cord continued throughout the life stages examined. Axolotls are still growing as adults and the PCNA labeling reflected that. There was no evidence of distinct zones of proliferation distinguishing intact stump cord and regenerating ependymal cell outgrowth in the adult, unlike the high PCNA labeling zone described by Rost et al. (2016) for larval cord after tail amputation. Axolotl ependymal cells in the regenerative outgrowth were proliferative and Msi-1 positive (**Figures 8F, 11E,F**), but intact adult Axolotl cord ependymal cells were proliferative and Msi-1 negative (**Figures 8D, 11E**). Msi-1 and proliferation are not irrevocably linked in the adult. Perhaps the low levels of Msi-2 are sufficient to maintain normal Notch signaling for the proliferation required in intact adult Axolotl CNS growth. Alternatively, the coexpression of Msi-1 and PCNA in regenerating adult tissue and *in vitro* may be a consequence of intracellular signaling triggered by mesenchymal ependymal organization (**Figures 8E, 10D, 13C**, rev. Kalluri and Weinberg, 2009).

### Msi and Tissue Organization

In Axolotl body region spinal cord, a clear epithelial/mesenchymal transition (EMT) process is involved in regenerative outgrowth (O’Hara et al., 1992; Chernoff et al., 2003). In *Xenopus* body cord gap replacement there is some disorganization, but the outgrowth assumes a more bulb-like organization, like that in newt cord or Axolotl tail amputation and the ampulla in *Xenopus* tail cord (rev. Chernoff, 1996; Clarke and Ferretti, 1998; Gargioli and Slack, 2004; Zukor et al., 2011).

The role of Msi expression in EMT or bulb outgrowth is not clear-cut. For example, Msi-1 and Msi-2 are overexpressed in a number of human carcinomas (cancers of epithelial origin), but downregulated in others (rev. Katz et al., 2014). In some cases Msi-1, -2 expression correlates with maintenance of epithelial organization and sometimes with EMT (Katz et al., 2014). In regenerating *Xenopus* and Axolotl spinal cord, Msi1 expression does not appear to be linked to epithelial organization, because it is strongly expressed during regenerative outgrowth *in vivo* and *in vitro* (**Figures 5, 6, 13**).

The pre-existing presence of Msi-1 (NF 50–53 *Xenopus*) vs. upregulation of Msi-1 (adult Axolotl) in regenerative ependymal outgrowth depends upon the life stage of the animal. The tadpoles are still developing; the adult Axolotl is mature. EGF (**Figure 12**) maintains the ependymal cells in a mesenchymal state in the cultures. Mesenchymally organized Axolotl ependymal cells always show Msi-1 expression (**Figure 13**). The EGF+ cultures also show that the regeneration-incompetent (NF 62+) *Xenopus* cells can no longer respond to conditions that maintain mesenchymal outgrowth. This correlates with the lower levels of Msi-1 seen in *Xenopus* NF 62 section *in situ* hybridization and semi-quantitative RT-PCR.

### Dorsal-ventral Patterning

#### Shh in *Xenopus*

In the intact NF 50–53 *Xenopus* cord and in the regenerated cord, Shh is coexpressed with Msi-1 in the floorplate (**Figures 7A,B,H,I**). The bulb-like regenerating outgrowth of the *Xenopus* cord lesion site maintained zones of gene expression along the dorsal-ventral axis with a Shh+ ventral zone largely complementary to Msi-1 outgrowth (**Figures 7F,G** and Supplementary Figure 6C). *Xenopus* dorsal-ventral axis expression of Msi-1 and Shh was coincident across the floorplate when a central canal was present in intact or reformed cord, and complementary only during regenerative outgrowth (**Figure 7** and Supplementary Figure 6). There were PCNA+ cells throughout the *Xenopus* regeneration tissue so the segregation of Msi-1 and Shh expression was not related to isolated zones of proliferating cells (**Figure 11C**).

Shh expression persists in the non-regenerating NF 62+ *Xenopus* cord (**Figure 7D**). The Shh is localized to the floorplate, which remains Msi-1 positive (**Figure 7C**).

#### Shh in Axolotl

In larval Axolotl tail cord, Shh expression is localized to the floorplate, as it is in higher vertebrate development (Schnapp et al., 2005). The very low levels of Shh mRNA in the intact adult Axolotl body region cord and strong upregulation after transection paralleled the pattern of Msi-1 mRNA and protein expression (**Figures 6, 9**). This reinforces the concept that the intact adult Axolotl cord is in a relatively stable, quiescent state developmentally that reactivates a developmental process after injury.

Work from other laboratories has shown that localized zones of Pax-6 and Pax-7 protein expression persist along the dorsal-ventral axis in intact adult Axolotl cord (Mchedlishvili et al., 2007). During spinal cord development, Delta/Notch signaling is upstream of Shh expression, which, in turn, regulates Pax gene expression in dorsal-ventral patterning (rev. Leigh and Maden, 2005). It remains to be seen whether the upregulation of Notch pathway associated Msi-1 expression acts upstream of Shh in the regenerating Axolotl cord.

The present study examined Msi family mRNA-binding protein expression known to be involved in neural stem cell behavior (Sakakibara et al., 1996; Good et al., 1998) in *Xenopus* and the Axolotl. What differs from other studies is the examination of (1) gap regeneration as opposed to amputation and unidirectional regeneration in (2) body region cord, not tail, where a combined cord and non-cord blastema forms, and (3) presents comparison of multiple life stages in *Xenopus* and the Axolotl. The choice of body region cord (above the *cauda equina*) makes comparison with mammalian cord injury models a little more direct than tail amputation studies. The use of adult Axolotl, where Msi-1 must be reactivated, is also more relevant to mammalian CNS injury responses.

## Ethics Statement

This study using amphibian animals was carried out according to NIH Guide under supervision of the IUPUI School of Science Institutional Animal Care and Use Committee (SARC).

## Author Contributions

All of the authors contributed to planning and execution of experiments. EC, HS, and DS executed the immunohistochemistry experiments. KS and TB-A executed the molecular biology studies. EC, HS, and KS executed the cell culture studies.

## Conflict of Interest Statement

The authors declare that the research was conducted in the absence of any commercial or financial relationships that could be construed as a potential conflict of interest.

## Footnotes

1 www.clustal.org

## References

[B1] Amamoto R. Huerta V. G. L. Takahashi E. Dai G. Grant A. K. Fu Z. (2016). Adult axolotls can regenerate original neuronal diversity in response to brain injury. *Elife* 5:e13998. 10.7554/eLife.13998 27156560 PMC4861602

[B2] Androutsellis-Theotokis A. Leker R. R. Soldner F. Hoeppner D. J. Ravin R. Poser S. W. (2006). Notch signalling regulates stem cell numbers *in vitro* and *in vivo*. *Nature* 442 823–826. 10.1038/nature04940 16799564

[B3] Barnabé-Heider F. Göritz C. Sabelström H. Takebayashi H. Pfrieger F. W. Meletis K. (2010). Origin of new glial cells in intact and injured adult spinal cord. *Cell Stem Cell* 7 470–482. 10.1016/j.stem.2010.07.014 20887953

[B4] Barrandon Y. Green H. (1987). Cell migration is essential for sustained growth of keratinocyte colonies: the roles of transforming growth factor-alpha and epidermal growth factor. *Cell* 50 1131–l137. 10.1016/0092-8674(87)90179-6 3497724

[B5] Battelli C. Nikopoulos G. N. Mitchell J. G. Verdi J. M. (2006). The RNA-binding protein Musashi-1 regulates neural development through the translational repression of p21(WAF-1). *Mol. Cell. Neurosci.* 31 85–96. 10.1016/j.mcn.2005.09.003 16214366

[B6] Beattie M. S. Bresnahan J. C. Lopate G. (1990). Metamorphosis alters the response to spinal cord transection in *Xenopus laevis* frogs. *J. Neurobiol.* 21 1108–1122. 10.1002/neu.480210714 2258724

[B7] Beck C. W. Christen B. Slack J. M. (2003). Molecular pathways needed for regeneration of spinal cord and muscle in a vertebrate. *Dev. Cell* 5 429–439. 10.1016/S1534-5807(03)00233-8 12967562

[B8] Becker C. G. Becker T. (2015). Neuronal regeneration from ependymo-radial glial cells: cook, little pot, cook. *Dev. Cell* 32 516–527. 10.1016/j.devcel.2015.01.001 25710537

[B9] Bodega G. Suarez I. Rubio M. Fernandez B. (1994). Ependyma: phylogenetic evolution of glial fibrillary acidic protein (GFAP) and vimentin expression in vertebrate spinal cord. *Histochemistry* 102 113–122. 10.1007/BF00269015 7822213

[B10] Bordzilovskaya N. P. Dettlaff T. A. Duhon S. T. (1989). “Developmental-stage series of Axolotl embryos,” in *Developmental Biology of the Axolotl* eds Armstrong J. B. Malacinski G. M. (New York, NY: Oxford University Press) 201–219.

[B11] Butler E. G. Ward M. B. (1965). Reconstitution of the spinal cord following ablation in urodele larvae. *J. Exp. Zool.* 160 47–66. 10.1002/jez.1401600106 5220031

[B12] Butler E. G. Ward M. B. (1967). Reconstitution of the spinal cord following ablation in adult *Triturus*. *Dev. Biol.* 15 464–486. 10.1016/0012-1606(67)90038-3 6032488

[B13] Chernoff E. A. Sato K. Corn A. Karcavich R. E. (2002). Spinal cord regeneration: intrinsic properties and emerging mechanisms. *Semin. Cell Dev. Biol.* 13 361–368. 10.1016/S1084952102000927 12324218

[B14] Chernoff E. A. G. (1996). Spinal cord regeneration: a phenomenon unique to urodeles? *Int. J. Dev. Biol.* 40 823–831. 8877457

[B15] Chernoff E. A. G. Henry L. C. Spotts T. (1998). An ependymal cell culture system for the study of spinal cord regeneration. *Wound Repair Regen.* 6 435–444. 10.1046/j.1524-475X.1998.60417.x 9824560

[B16] Chernoff E. A. G. Munck C. M. Egar M. W. Mendelsohn L. G. (1990). Primary cultures of axolotl spinal cord ependymal cells. *Tissue Cell* 5 601–613. 10.1016/0040-8166(90)90058-H 18620322

[B17] Chernoff E. A. G. O’Hara C. M. Bauerle D. Bowling M. (2000). Matrix metalloproteinase production in regenerating axolotl spinal cord. *Wound Repair Regen.* 8 282–291. 10.1046/j.1524-475x.2000.00282.x 11013020

[B18] Chernoff E. A. G. Stocum D. L. Nye H. L. D. Cameron J. A. (2003). Urodele spinal cord regeneration and related processes. *Dev. Dyn.* 226 295–307. 10.1002/dvdy.10240 12557207

[B19] Clarke J. D. Tonge D. A. Holder N. H. (1986). Stage-dependent restoration of sensory dorsal columns following spinal cord transection in anuran tadpoles. *Proc. R. Soc. Lond. B Biol. Sci.* 227 67–82. 10.1098/rspb.1986.0010 2870501

[B20] Clarke J. D. W. Ferretti P. (1998). “CNS regeneration in lower vertebrates,” in *Cellular and Molecular Basis of Regeneration: from Invertebrates to Humans* eds Ferretti P. Geraudie J. (Chichester: John Wiley and Sons, Ltd.) 255–269.

[B21] Clarke N. B. (1983). Evolution of calcium regulation in lower vertebrates. *Am. Zool.* 23 719–727. 10.1093/icb/23.3.719

[B22] Davis B. M. Duffy M. T. Simpson S. B. Jr. (1989). Bulbospinal and intraspinal connections in normal and regenerated salamander spinal cord. *Exp. Neurol.* 103 41–51. 10.1016/0014-4886(89)90183-0 2912749

[B23] Dent J. N. (1962). Limb regeneration in larvae and metamorphosing individuals of the South African clawed toad. *J. Morphol.* 110 61–77. 10.1002/jmor.1051100105 13885494

[B24] Detwiler S. R. (1947). Restitution of the brachial region of the cord following unilateral excision in the. (embryo). *J. Exp. Zool.* 104 53–68. 10.1002/jez.1401040104 20285007

[B25] Diaz Quiroz J. F. Echeverri K. (2013). Spinal cord regeneration: where fish, frogs and salamanders lead the way, can we follow? *Biochem. J.* 451 353–364. 10.1042/BJ20121807 23581406

[B26] Egar M. Singer M. (1972). The role of ependyma in spinal cord regeneration in the urodele, *Triturus*. *Exp. Neurol.* 37 422–430. 10.1016/0014-4886(72)90085-4 4637959

[B27] Ehm O. Goritz C. Covic M. Schaffner I. Schwarz T. J. Karaca E. (2010). RBPJ kappa-dependent signaling is essential for long-term maintenance of neural stem cells in the adult hippocampus. *J. Neurosci.* 30 13794–13807. 10.1523/JNEUROSCI.1567-10.2010 20943920 PMC6633732

[B28] Faigle R. Song H. (2013). Signaling mechanisms regulating adult neural stem cells and neurogenesis. *Biochim. Biophys. Acta* 1830 2435–2448. 10.1016/j.bbagen.2012.09.002 22982587 PMC3541438

[B29] Feder M. E. Burggren W. W. (eds) (1992). *Environmental Physiology of the Amphibians.* Chicago, IL: University of Chicago Press.

[B30] Fei J.-F. Schuez M. Tazaki A. Taniguchi Y. Roensch K. Tanaka E. M. (2014). CRISPR-mediated genomic deletion of *Sox*2 in the axolotl shows a requirement in spinal cord neural stem cell amplification during tail regeneration. *Stem Cell Rep.* 3 444–459. 10.1016/j.stemcr.2014.06.018 25241743 PMC4266004

[B31] Ferre F. (1992). Quantitative or semi-quantitative PCR: reality versus myth. *PCR Methods Appl.* 2 1–9. 10.1101/gr.2.1.1 1490169

[B32] Ferretti P. Zhang F. O’Neill P. (2003). Changes in spinal cord regenerative ability through phylogenesis and development: lessons to be learnt. *Dev. Dyn.* 226 245–256. 10.1002/dvdy.10226 12557203

[B33] Forehand C. J. Farel P. B. (1982). Anatomical and behavioral recovery from the effects of spinal cord transection: dependence on metamorphosis in anuran larvae. *J. Neurosci.* 2 652–654. 6176699 10.1523/JNEUROSCI.02-05-00654.1982 PMC6564260

[B34] Friedl P. Gilmour D. (2009). Collective cell migration in morphogenesis, regeneration and cancer. *Nat. Rev. Mol. Cell Biol.* 10 445–457. 10.1038/nrm2720 19546857

[B35] Gaete M. Muñoz R. Sánchez N. Tampe R. Moreno M. Contreras E. G. (2012). Spinal cord regeneration in *Xenopus tadpoles* proceeds through activation of Sox2-positive cells. *Neural Dev.* 7:13. 10.1186/1749-8104-7-13 22537391 PMC3425087

[B36] Gargioli C. Slack J. M. (2004). Cell lineage tracing during *Xenopus* tail regeneration. *Development* 131 2669–2679. 10.1242/dev.01155 15148301

[B37] Gervasi C. Stewart C. B. Szaro G. G. (2000). *Xenopus laevis* peripherin (XIF3) is expressed in radial glia and proliferating neural epithelial cells as well as in neurons. *J. Comp. Neurol.* 423 512–531. 10.1002/1096-9861(20000731)423:3<512::AID-CNE13>3.0.CO;2-1 10870090

[B38] Good P. Yoda A. Sakakibara S. Yamamoto A. Imai T. Sawa H. (1998). The human Musashi homolog 1 (MSI1) gene encoding the homologue of Musashi/Nrp-1 a neural RNA-binding protein putatively expressed in CNS stem cells and neural progenitor cells. *Genomics* 52 382–384. 10.1006/geno.1998.5456 9790759

[B39] Good P. J. Rebbert M. L. Dawid I. B. (1993). Three new members of the RNP protein family in *Xenopus*. *Nucleic Acids Res.* 21 999–1006. 10.1093/nar/21.4.999 8451200 PMC309235

[B40] Henikoff S. Henikoff J. G. (1992). Amino acid substitution matrices from protein blocks (amino add sequence/alignment algorithms/data base searching. *Proc. Natl. Acad. Sci. U.S.A.* 89 10915–10919. 10.1073/pnas.89.22.10915 1438297 PMC50453

[B41] Holder N. Clarke J. D. W. Wilson S. Hunter K. Tonge D. A. (1989). “Mechanisms controlling directed axon regeneration in the peripheral and central nervous systems of amphibians,” in *NATO Advanced Research Workshop on Recent Trends in Regeneration Research (1988: Athens, Greece)* eds Kiorstsis V. Koussoulakos S. Wallace H. (New York: Plenum Press) 179–190.

[B42] Holtzer H. (1951). Reconstitution of the urodele spinal cord following unilateral ablation. *J. Exp. Zool.* 117 523–558. 10.1002/jez.1401170308

[B43] Holtzer H. (1952). Reconstitution of the urodele spinal cord following unilateral ablation. *J. Exp. Zool.* 119 263–301. 10.1002/jez.1401190205

[B44] Horisawa K. Imai T. Okano H. Yanagawa H. (2010). The Musashi family RNA-binding proteins in stem cells. *Biomol. Concepts* 1 59–66. 10.1515/bmc.2010.005 25961986

[B45] Horky L. L. Galimi F. Gage F. H. Horner P. J. (2006). Fate of endogenous stem/progenitor cells following spinal cord injury. *J. Comp. Neurol.* 498 525–538. 10.1002/cne.21065 16874803 PMC2553041

[B46] Imai T. Tokunaga A. Yoshida T. Hashimoto M. Mikoshiba K. Weinmaster G. (2001). The neural RNA-binding protein Musashi1 translationally regulates mammalian numb gene expression by interacting with its mRNA. *Mol. Cell. Biol.* 21 3888–3900. 10.1128/MCB.21.12.3888-3900.2001 11359897 PMC87052

[B47] Jiménez A. J. Domínguez-Pinos M.-D. Guerra M. M. Fernández-Llebrez P. Pérez-Fígares J.-M. (2014). Structure and function of the ependymal barrier and diseases associated with ependyma disruption. *Tissue Barriers* 2:e28426. 10.4161/tisb.28426 25045600 PMC4091052

[B48] Kalluri R. Weinberg R. A. (2009). The basics of epithelial-mesenchymal transition. *J. Clin. Invest.* 119 1420–1428. 10.1172/JCI39104 19487818 PMC2689101

[B49] Kaneko Y. Sakakibara S. Imai T. Suzuki A. Nakamura Y. Sawamoto K. (2000). Musashi1: an evolutionally conserved marker for CNS progenitor cells including neural stem cells. *Dev. Neurosci.* 22 139–153. 10.1159/000017435 10657706

[B50] Katz Y. Li F. Lambert N. J. Sokol E. S. Tam W. L. Cheng A. W. (2014). Musashi proteins are post-transcriptional regulators of the epithelial-luminal cell state. *Elife* 3:e03915. 10.7554/eLife.03915 25380226 PMC4381951

[B51] Keinath M. C. Timoshevskaya N. Y. Timoshevskiy V. A. Tsonis P. A. Voss S. R. Smith J. J. (2015). Initial characterization of the large genome of the salamander *Ambystoma mexicanum* using shotgun and laser capture chromosome sequencing. *Sci. Rep.* 5:16413. 10.1038/srep16413 26553646 PMC4639759

[B52] Kojima A. Tator C. H. (2002). Intrathecal administration of epidermal growth factor and fibroblast growth factor 2 promotes ependymal proliferation and functional recovery after spinal cord injury in adult rats. *J. Neurotrauma* 19 223–238. 10.1089/08977150252806974 11893024

[B53] Lacroix S. Hamilton L. K. Vaugeois A. Beaudoin S. Breault-Dugas C. Pineau I. (2014). Central canal ependymal cells proliferate extensively in response to traumatic spinal cord injury but not demyelinating lesions. *PLoS One* 9:e85916. 10.1371/journal.pone.0085916 24475059 PMC3903496

[B54] Lee-Liu D. Edwards-Faret G. Tapia V. S. Larraín J. (2013). Spinal cord regeneration: lessons for mammals from non-mammalian vertebrates. *Genesis* 51 529–544. 10.1002/dvg.22406 23760835

[B55] Leigh W. Maden M. (2005). The mechanisms of dorsoventral patterning in the vertebrate neural tube. *Dev. Biol.* 282 1–13. 10.1016/j.ydbio.2005.02.027 15936325

[B56] Li X. Floriddia E. M. Toskas K. Fernandes K. J. L. Guérout N. Barnabé-Heider F. (2016). Regenerative potential of ependymal cells for spinal cord injuries over time. *EBioMedicine* 13 55–65. 10.1016/j.ebiom.2016.10.035 27818039 PMC5264475

[B57] Maier C. E. Miller R. N. (1997). Notochord is essential for oligodendrocyte development in *Xenopus* spinal cord. *J. Neurosci. Res.* 47 361–371. 10.1002/(SICI)1097-4547(19970215)47:4<361::AID-JNR1>3.0.CO;2-C 9057129

[B58] Makanae A. Mitogawa K. Satoh A. (2016). Cooperative inputs of Bmp and Fgf signaling induce tail regeneration in urodele amphibians. *Dev. Biol.* 410 45–55. 10.1016/j.ydbio.2015.12.012 26703427

[B59] Martinez-De Luna R. I. Ku R. Y. Aruck A. M. Santiago F. Viczian A. S. San Mauro D. (2017). Müller glia reactivity follows retinal injury despite the absence of the glial fibrillary acidic protein gene in *Xenopus*. *Dev. Biol.* 426 219–235. 10.1016/j.ydbio.2016.03.005 26996101 PMC5026855

[B60] Mchedlishvili L. L. Epperlein H. H. Anja Telzerow A. Tanaka E. M. (2007). A clonal analysis of neural progenitors during axolotl spinal cord regeneration reveals evidence for both spatially restricted and multipotent progenitors. *Development* 134 2083–2093. 10.1242/dev.02852 17507409

[B61] Meletis K. Barnabé-Heider F. Carlén M. Evergren E. Tomilin N. Shupliakov O. (2008). Spinal cord injury reveals multilineage differentiation of ependymal cells. *PLoS Biol.* 6:e182. 10.1371/journal.pbio.0060182 18651793 PMC2475541

[B62] Mitashov V. I. Maliovanova S. D. (1982). Cellular proliferative potentials of the pigment and ciliated epithelium of the eye in clawed toads normally and during regeneration. *Ontogenez* 13 228–234. 7099515

[B63] Monaghan J. R. Walker J. A. Beachy C. K. Voss S. R. (2007). Early gene expression during natural spinal cord regeneration in the salamander *Ambystoma mexicanum*. *J. Neurochem.* 101 27–40. 10.1111/j.1471-4159.2006.04344.x 17241119

[B64] Moore S. A. (2016). The spinal ependymal layer in health and disease. *Vet. Pathol.* 53 746–753. 10.1177/0300985815618438 26792842

[B65] Mothe A. J. Tator C. H. (2005). Proliferation, migration, and differentiation of endogenous ependymal region stem/progenitor cells following minimal spinal cord injury in the adult rat. *Neuroscience* 13 177–187. 10.1016/j.neuroscience.2004.10.011 15680701

[B66] Muñoz R. Edwards-Fareta G. Morenoa M. Zuñigaa N. Cline H. Larrain J. (2015). Regeneration of *Xenopus laevis* spinal cord requires Sox2/3 expressing cells. *Dev. Biol.* 408 229–243. 10.1016/j.ydbio.2015.03.009 25797152 PMC4826040

[B67] Nakamura M. Okano H. Blendy J. A. Montell C. (1994). Musashi, a neural RNA-binding protein required for Drosophila adult external sensory organ development. *Neuron* 13 67–81. 10.1016/0896-6273(94)90460-X 8043282

[B68] Nieuwkoop P. D. Faber J. (1956). *Normal Table of Xenopus laevis (Daudin); a Systematical and Chronological Survey of the Development from the Fertilized Egg Till the End of Metamorphosis.* Amsterdam: North-Holland Pub Co. 243.

[B69] Nowoshilow S. Schloissnig S. Fei J. F. Dahl A. Pang A. W. C. Pippel M. (2018). The axolotl genome and the evolution of key tissue formation regulators. *Nature* 554 50–55. 10.1038/nature25458 29364872

[B70] O’Hara C. M. Chernoff E. A. G. (1994). Growth factor modulation of injury-reactive ependymal cell proliferation and migration. *Tissue Cell* 26 599–611. 10.1016/0040-8166(94)90012-4 8091423

[B71] O’Hara C. M. Egar M. W. Chernoff E. A. (1992). Reorganization of the ependyma during axolotl spinal cord regeneration: changes in intermediate filament and fibronectin expression. *Dev. Dyn.* 193 103–115. 10.1002/aja.1001930202 1374657

[B72] Oksche A. Ueck M. (1976). “The nervous system. Ch.7” in *Physiology of the Amphibia* Vol. 3 ed. Lofts B. (New York, NY: Academic Press) 314–420. 10.1016/B978-0-12-455403-0.50012-8

[B73] Panayiotou E. Malas S. (2013). Adult spinal cord ependymal layer: a promising pool of quiescent stem cells to treat spinal cord injury. *Front. Physiol.* 4:340. 10.3389/fphys.2013.00340 24348422 PMC3842874

[B74] Pannese E. (2015). “The Neuroglia of the CNS Ch. VIII,” in *Neurocytology: Fine Structure of Neurons, Nerve Processes, and Neuroglial Cells* 2nd Edn (Switzerland: Springer) 183–224.

[B75] Park J. H. Han H. J. (2009). Caveolin-1 plays important role in EGF-induced migration and proliferation of mouse embryonic stem cells: involvement of PI3K/Akt and ERK. *Am. J. Physiol. Cell Physiol.* 297 C935–C944. 10.1152/ajpcell.00121.2009 19625610

[B76] Piatt J. (1955). “Regeneration in the central nervous system of amphibia,” in *Regeneration in the Central Nervous System* ed. Windle W. F. (Springfield, IL: C. Thomas Publishing) 20–46.

[B77] Ratti A. Fallini C. Cova L. Fantozzi R. Calzarossa C. Zennaro E. (2006). A role for the ELAV RNA-binding proteins in neural stem cells: stabilization of Msi1 mRNA. *J. Cell Sci.* 119 1442–1452. 10.1242/jcs.02852 16554442

[B78] Reichenbach A. Wolberg H. (2013). “Astrocytes and ependymal glia. Section 1. Article 4” in *Neuroglia* 3rd Edn eds Helmut Kettenmann H. Ransom B. R. (Oxford: Oxford Press) 35–49.

[B79] Ren Y. Ao Y. O’Shea T. M. Burda J. E. Bernstein A. M. Brumm A. J. (2017). Ependymal cell contribution to scar formation after spinal cord injury is minimal, local and dependent on direct ependymal injury. *Nat. Sci. Rep.* 7:41122. 10.1038/srep41122 28117356 PMC5259707

[B80] Richter K. Good P. J. Dawid I. B. (1990). A developmentally regulated, nervous system-specific gene in *Xenopus* encodes a putative RNA-binding protein. *New Biol.* 2 556–565. 1708282

[B81] Roberts A. (2000). Early functional organization of spinal neurons in developing lower vertebrates. *Brain Res. Bull.* 53 585–593. 10.1016/S0361-9230(00)00392-0 11165794

[B82] Rost F. Rodrigo Albors A. Mazurov V. Brusch L. Deutsch A. Tanaka E. M. (2016). Accelerated cell divisions drive the outgrowth of the regenerating spinal cord in axolotls. *Elife* 5:e20357. 10.7554/eLife20357 27885987 PMC5182066

[B83] Sakakibara S. Imai T. Hamaguchi K. Okabe M. Aruga J. Nakajima K. (1996). Mouse-Musashi-1 a neural RNA-binding protein highly enriched in the mammalian CNS stem cell. *Dev. Biol.* 176 230–242. 10.1006/dbio.1996.0130 8660864

[B84] Sakakibara S.-I. Nakamura Y. Satoh H. Okano H. (2001). RNA-binding protein musashi2: developmentally regulated expression in neural precursor cells and subpopulations of neurons in mammalian CNS. *J. Neurosci.* 21 8091–8107. 11588182 10.1523/JNEUROSCI.21-20-08091.2001 PMC6763847

[B85] Sarria D. A. Nguyen H. V. Egar M. W. Chernoff E. A. G. (2008). Meningeal organization and injury response in amphibian spinal cord regeneration. *Dev. Biol.* 319 553–560. 10.1016/j.ydbio.2008.05.325

[B86] Sato K. Yasugi S. (1997). Chicken keratin-19: Cloning of cDNA and analysis of expression in the chicken embryonic gut. *Dev. Growth Differ.* 39 751–761. 10.1046/j.1440-169X.1997.t01-5-00011.x 9493835

[B87] Savagner P. (2001). Leaving the neighborhood: molecular mechanisms involved during epithelial-mesenchymal transition. *BioEssays* 23 912–923. 10.1002/bies.1132 11598958

[B88] Schnapp E. Kragl M. Rubin L. Tanaka E. M. (2005). Hedgehog signaling controls dorsoventral patterning, blastema cell proliferation and cartilage induction during Axolotl tail regeneration. *Development* 132 3243–3253. 10.1242/dev.01906 15983402

[B89] Shi Y.-B. (2000). *Amphibian Metamorphosis. From Morphology to Molecular Biology.* New York, NY: John Wiley and Sons 135–153.

[B90] Smith J. J. Putta S. Walker J. A. Kump D. K. Samuels A. K. Monaghan J. R. (2005). Sal-Site: integrating new and existing ambystomatid salamander research and informational resources. *BMC Genomics* 6:181. 10.1186/1471-2164-6-181 16359543 PMC1351182

[B91] Stensaas L. J. (1983). “Regeneration in the spinal cord of the newt *Notophthalmus* (*Triturus*) *pyrrhogaster*,” in *Spinal Cordreconstruction* eds Kao C. C. Bunge R. P. Reier P. J. (New York, NY: Raven Press) 121–149.

[B92] Tanaka E. M. Ferretti P. (2009). Considering the evolution of regeneration in the central nervous system. *Nat. Rev. Neurosci.* 10 713–723. 10.1038/nrn2707 19763104

[B93] Tazaki A. Tanaka E. M. Fe J.-F. (2017). Salamander spinal cord regeneration: the ultimate positive control in vertebrate spinal cord regeneration. *Dev. Biol.* 432 63–71. 10.1016/j.ydbio.2017.09.034 29030146

[B94] Veldhoen N. Crump D. Werry K. Helbing C. C. (2002). Distinctive gene profiles occur at key points during natural metamorphosis in the *Xenopus laevis* tadpole tail. *Dev. Dyn.* 225 457–468. 10.1002/dvdy.10175 12454923

[B95] Walder S. Zhang F. Ferretti P. (2003). Up-regulation of neural stem cell markers suggests the occurrence of dedifferentiation in regenerating spinal cord. *Dev. Genes. Evol.* 213 625–630. 10.1007/s00427-003-0364-2 14608505

[B96] Wells A. Ware M. F. Allen F. D. Lauffenburger D. A. (1999). Shaping up for shipping out: PLCg signaling of morphology changes in EGF-stimulated fibroblast migration. *Cell Motil. Cytoskeleton* 44 227–233. 10.1002/(SICI)1097-0169(199912)44:4<227::AID-CM1>3.0.CO;2-B 10602252

[B97] Wilkinson D. G. (1992). “Whole mount in situ hybridization of vertebrate embryos,” in *In Situ Hybridization: A Practical Approach* ed. Wilson D. G. (Oxford: IRL Press) 75–83.

[B98] Wilkinson D. G. (1993). “*In situ* hybridization,” in *Essential Developmental Biology, a Practical Approach* eds Stern C. D. Holland P. W. H. (Oxford: IRL Press) 257–274.

[B99] Zhang F. Clarke J. D. W. Ferretti P. (2000). FGF-2 up-regulation and proliferation of neural progenitors in the regenerating amphibian spinal cord. *Dev. Biol.* 225 381–391. 10.1006/dbio.2000.9843 10985857

[B100] Zukor K. A. Kent D. T. Odelberg S. J. (2011). Meningeal cells and glia establish a permissive environment for axon regeneration after spinal cord injury in newts. *Neural Dev.* 6:1. 10.1186/1749-8104-6-1 21205291 PMC3025934

