# Supplementary figures and images for: Musashi and Plasticity of Xenopus and Axolotl Spinal Cord Ependymal Cells
Source: Front Cell Neurosci. 2018 Feb 27;12:45. doi: 10.3389/fncel.2018.00045 (PMC5835034; doi:10.3389/fncel.2018.00045)

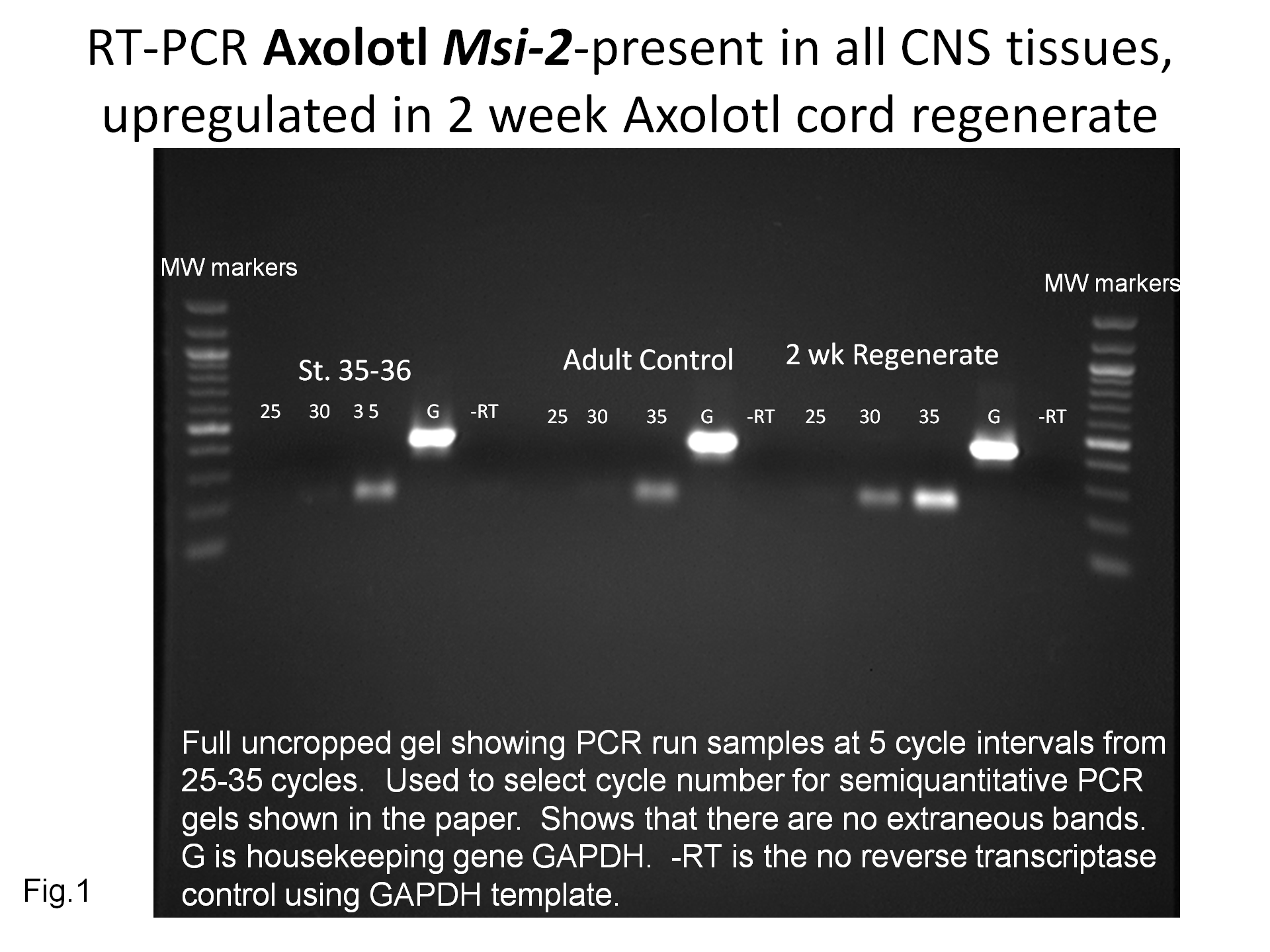

Supplement: Supplementary file 1 [file Image_1.TIF]

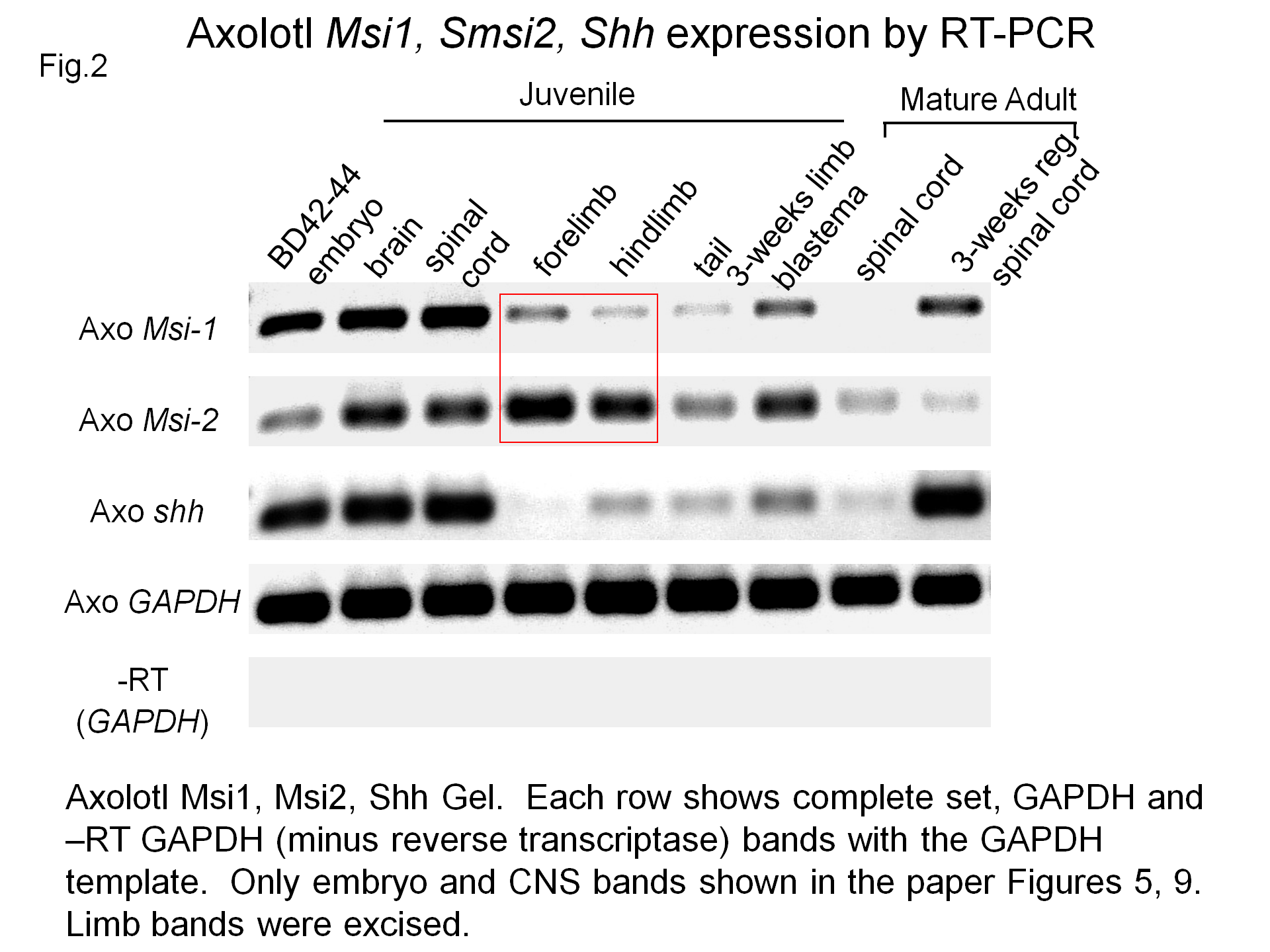

Supplement: Supplementary file 2 [file Image_2.TIF]

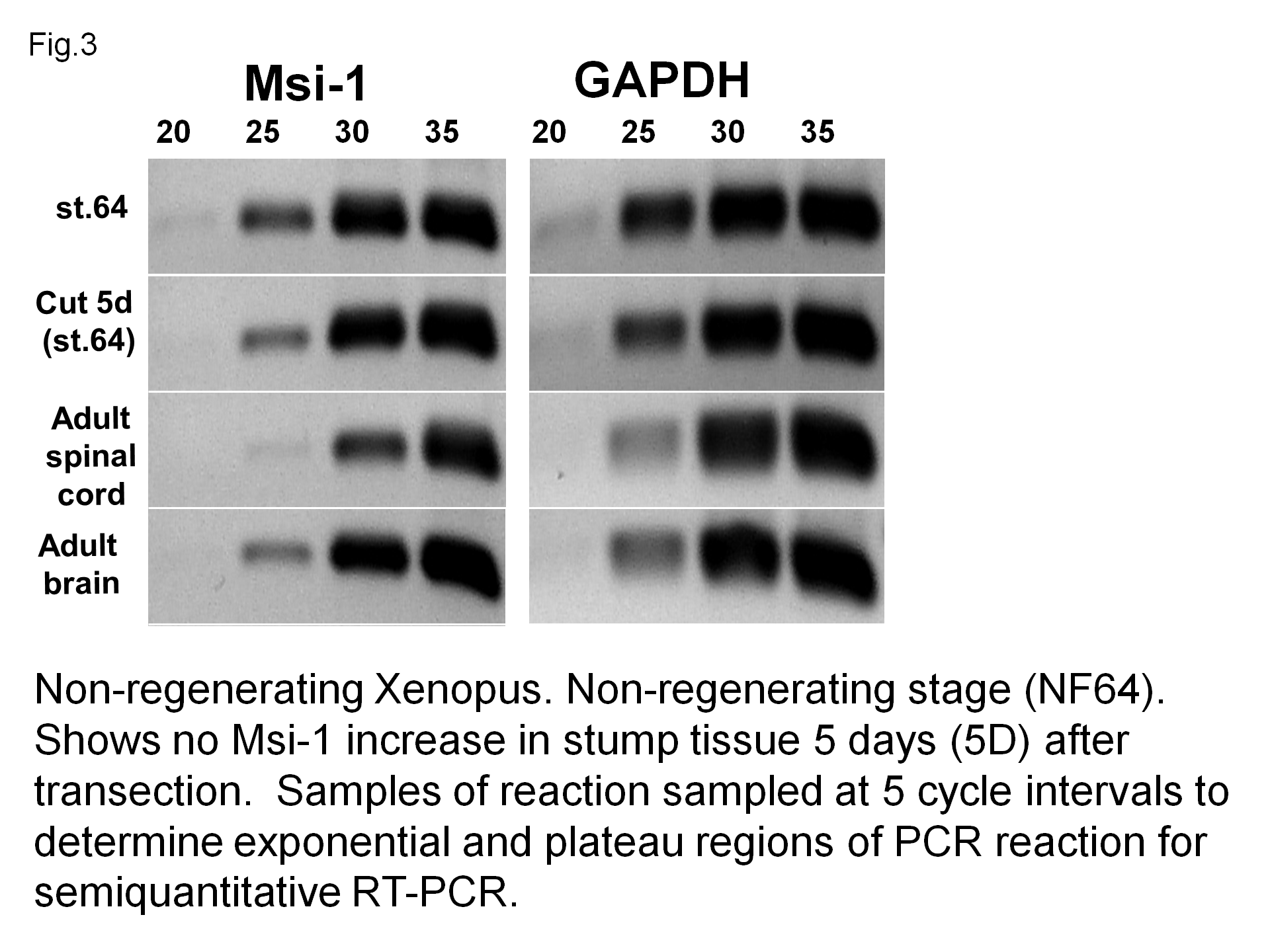

Supplement: Supplementary file 3 [file Image_3.TIF]

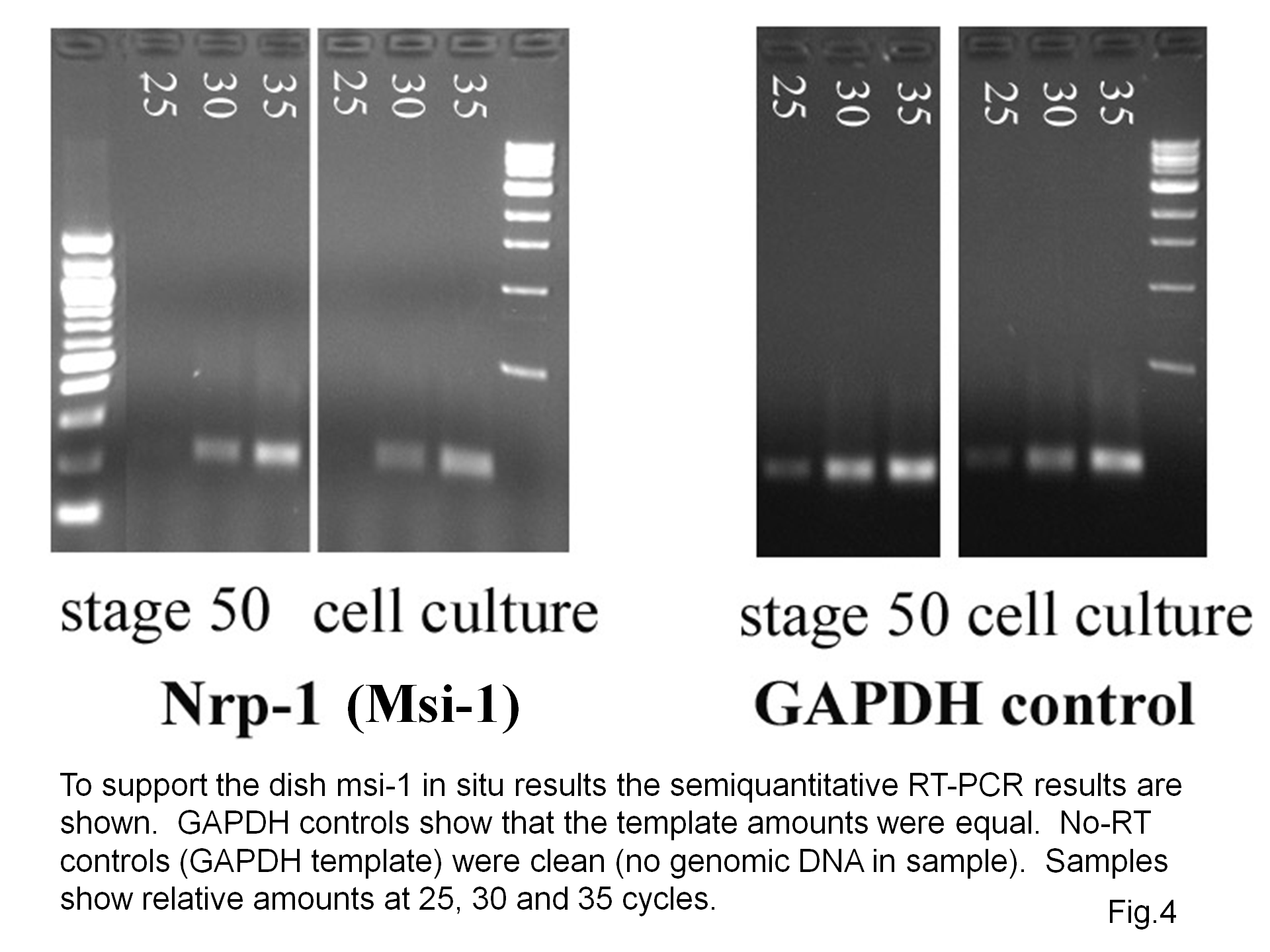

Supplement: Supplementary file 4 [file Image_4.TIF]

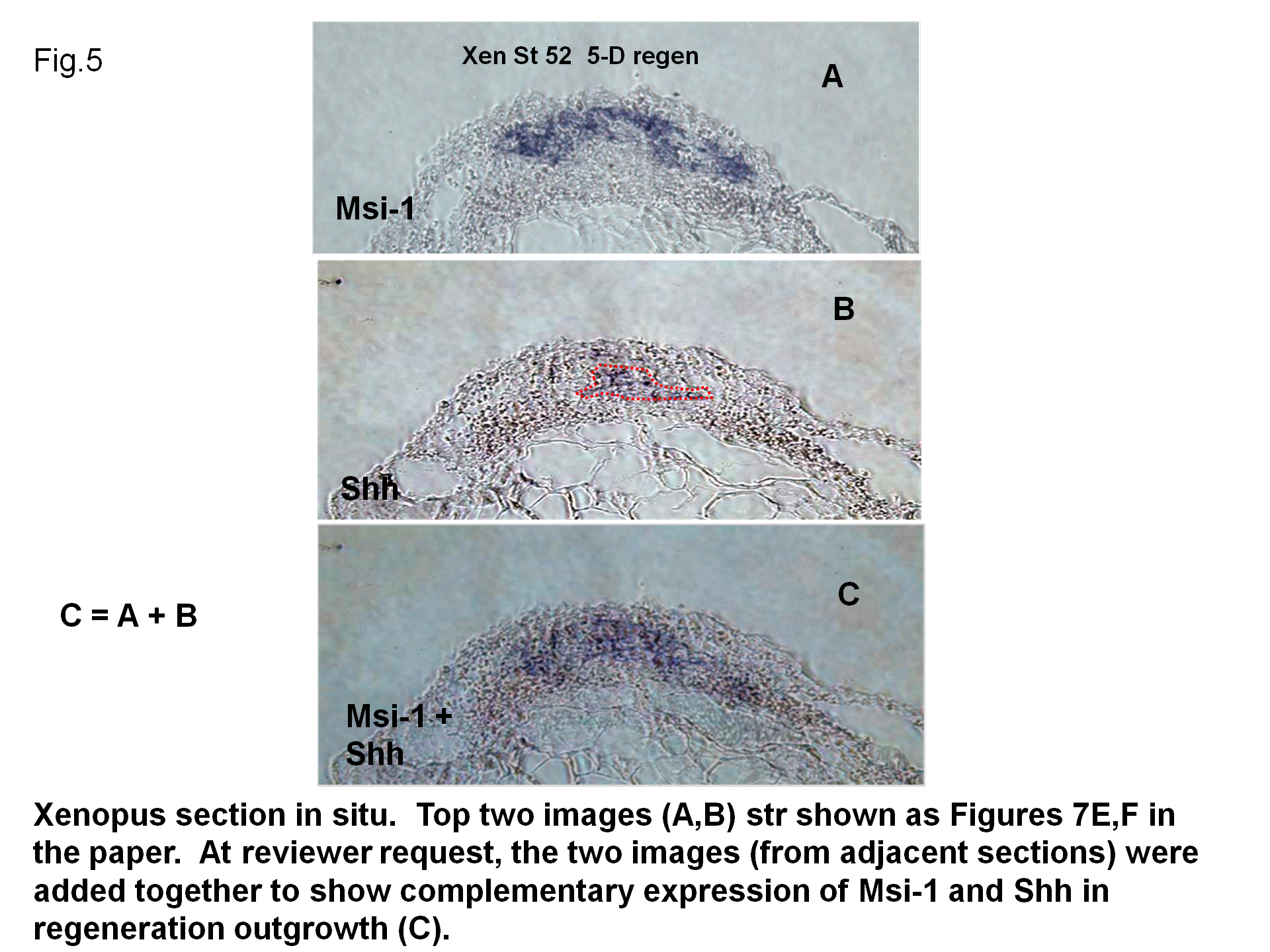

Supplement: Supplementary file 5 [file Image_5.TIF]

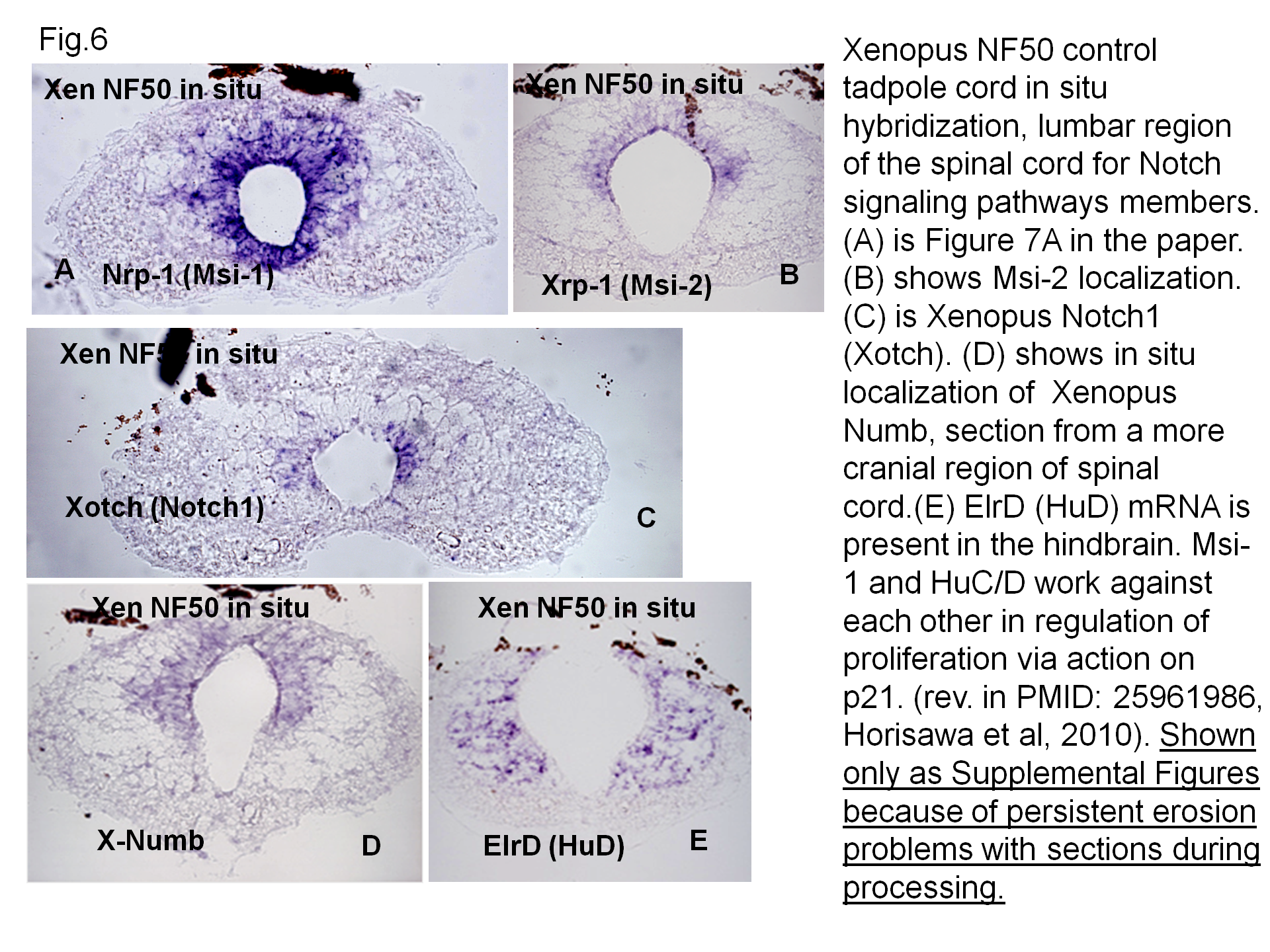

Supplement: Supplementary file 6 [file Image_6.TIF]

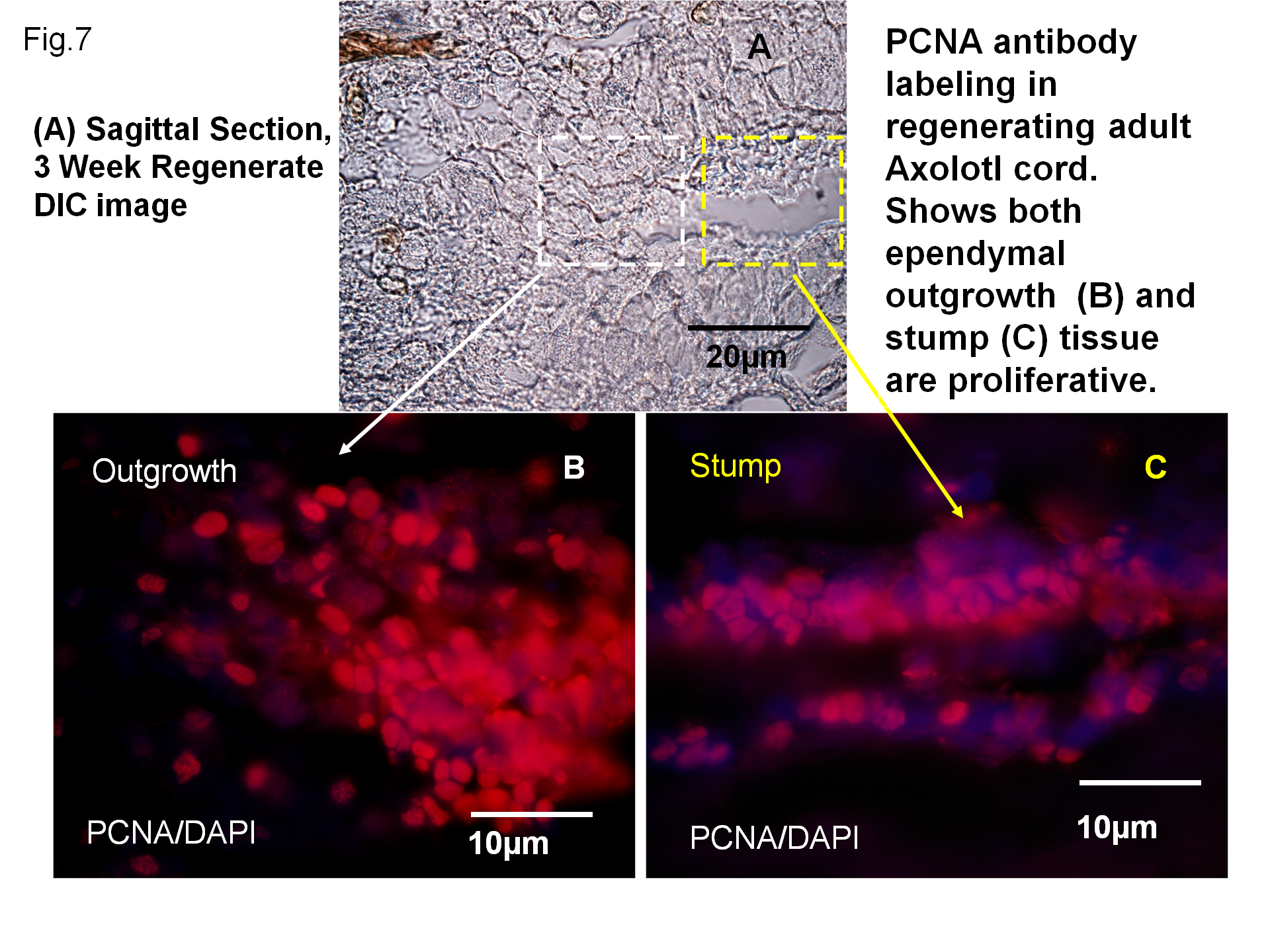

Supplement: Supplementary file 7 [file Image_7.TIF]

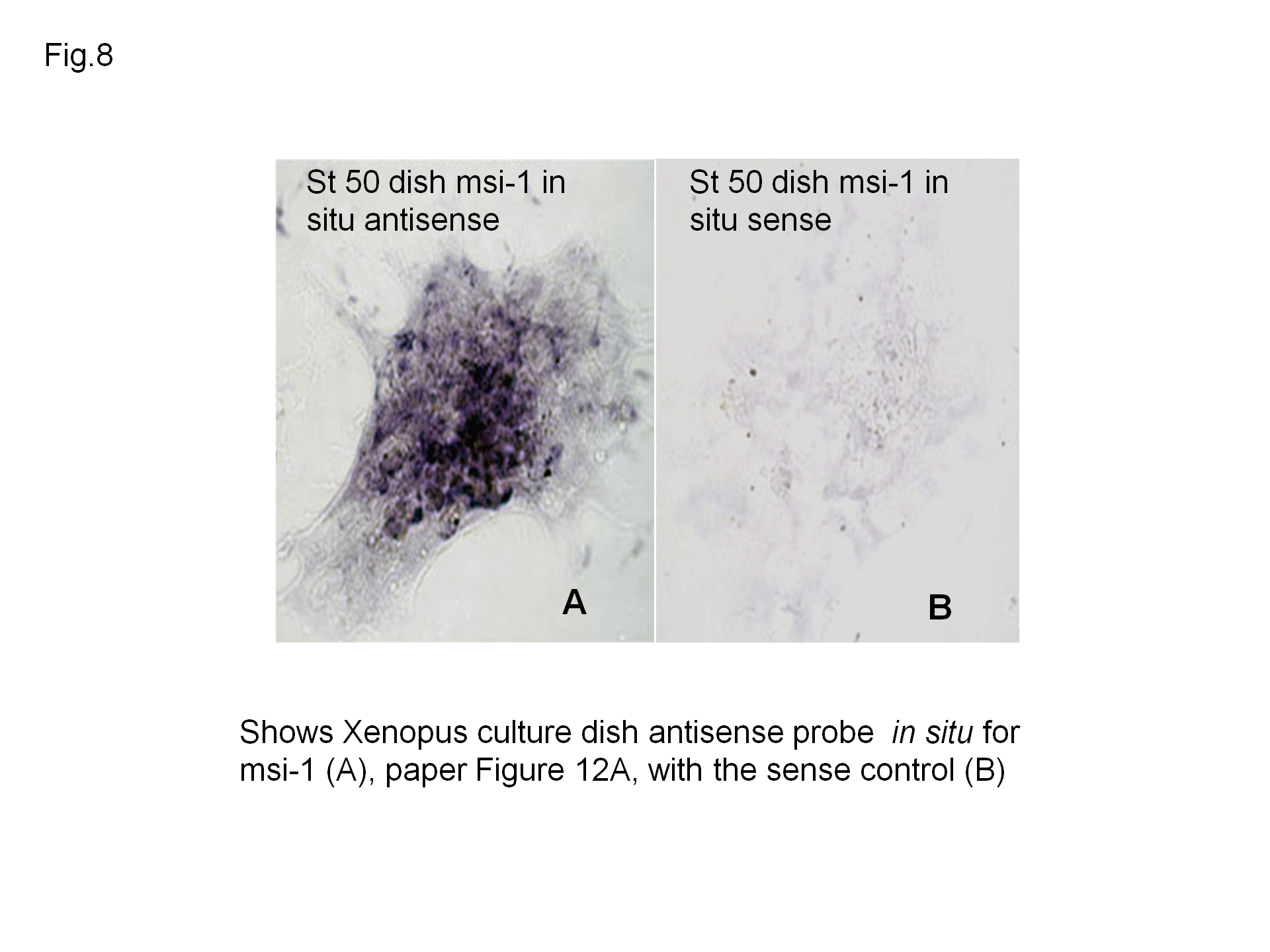

Supplement: Supplementary file 8 [file Image_8.TIF]
